# The *SPL8‐YAB3/TCP19* module integrates transcriptional and protein–protein regulatory mechanisms to orchestrate anther development in Arabidopsis

**DOI:** 10.1111/tpj.71160

**Published:** 2026-10-11

**Authors:** Tingting Li, Lei Zhang, Shuping Xing

**Affiliations:** ^1^ School of Life Science Shanxi University Taiyuan 030006 China; ^2^ Institute of Applied Biology Shanxi University Taiyuan 030006 China; ^3^ Faculty of Biology and Biotechnology Ruhr University Bochum Bochum 44801 Germany; ^4^ Shanxi Key Laboratory of Nucleic Acid Biopesticides Shanxi University Taiyuan 030600 China; ^5^ Present address: Chongqing Technology and Business University Chongqing 400072 China

**Keywords:** *SPL8*–*YAB3*/*TCP19* regulatory module, yeast two‐hybrid (Y2H), bimolecular fluorescence complementation (BiFC), FRET‐FLIM, ChIP‐qPCR, yeast one‐hybrid (Y1H), dual‐luciferase assay, anther development, *Arabidopsis thaliana*

## Abstract

SQUAMOSA PROMOTER BINDING PROTEIN‐LIKE 8 (SPL8) is a plant‐specific transcription factor essential for anther development, but its regulatory partners and downstream mechanisms remain largely unknown. We identified 26 SPL8‐interacting transcription factors by yeast two‐hybrid screening and bimolecular fluorescence complementation (BiFC) assays, with YABBY (YAB) and TEOSINTE BRANCHED 1/CYCLOIDEA/PCF (TCP) family emerging as the predominant interactors. Among these, YAB3 and TCP19 were selected for detailed characterization. Their interactions with SPL8, as well as with each other, were confirmed in plant nuclei by fluorescence resonance energy transfer–fluorescence lifetime imaging microscopy (FRET‐FLIM). Chromatin immunoprecipitation sequencing (ChIP‐seq), ChIP‐qPCR, yeast one‐hybrid, and dual‐luciferase assays demonstrated that SPL8 directly binds the regulatory regions of *YAB3* and *TCP19* and activates their transcription. Dexamethasone‐inducible expression assays further showed that this induction occurs independently of de novo protein synthesis. Expression analyses revealed overlapping spatial and temporal patterns of *SPL8*, *YAB3*, and *TCP19* during early anther development, particularly in tapetal cells, microspore mother cells, and vascular tissues. Genetic and cellular analyses of promoter‐driven SRDX repression lines showed that *SPL8*, *YAB3*, and *TCP19* regulate anther morphogenesis, tapetal differentiation, meiotic progression, and microspore development. Although individual family members exhibited functional redundancy, their repression caused severe developmental defects and male sterility. Together, these findings establish the *SPL8*–*YAB3*/*TCP19* module as a multilayered regulatory system in which *SPL8* controls anther development through both transcriptional activation of *YAB3* and *TCP19* and physical interaction with these proteins, expanding the regulatory framework of *SPL*‐mediated reproductive development.

## Significance Statement

*SQUAMOSA PROMOTER BINDING PROTEIN‐LIKE 8* (*SPL8*) is essential for plant reproduction, yet its downstream targets remain largely unknown. We identify a previously unrecognized *SPL8*–*YAB3*/*TCP19* regulatory module in which *SPL8* directly regulates the expression of *YAB3* and *TCP19,* while also physically interacting with their proteins during anther development, thereby unveiling a multi‐tiered regulatory network that substantially expands the current understanding of *SPL*‐mediated reproductive control.

## INTRODUCTION

The development of functional male gametophytes in flowering plants is essential for sexual reproduction and crop yield. In *Arabidopsis thaliana*, anther development follows a precisely ordered program that depends on the specification, differentiation, and coordinated development of multiple somatic and reproductive cell lineages (Chang & Ma, 2026; Sanders et al., 1999; Scott et al., 2004). This process is controlled by complex transcriptional regulatory networks, in which several core factors, including *SPOROCYTELESS*/*NOZZLE* (*SPL*/*NZZ*), *EXCESS MICROSPOROCYTES 1*/*EXTRA SPOROGENOUS CELLS* (*EMS1*/*EXS*), *T* *APETUM DETERMINANT 1* (*TPD1*), *SOMATIC EMBRYOGENESIS RECEPTOR KINASE1*/*2* (*SERK1*/*2*), *DYSFUNCTIONAL TAPETUM1* (*DYT1*), *DEFECTIVE IN TAPETAL DEVELOPMENT AND FUNCTION 1* (*TDF1*), and *ABORTED MICROSPORES* (*AMS*), regulate key signaling cascades (Canales et al., 2002; Colcombet et al., 2005; Schiefthaler et al., 1999; Sorensen et al., 2003; Verma, 2019; Yang et al., 1999; Zhang et al., 2006; Zhao et al., 2002; Zhu et al., 2008).

In addition to these core regulators, several other gene families—including SBP‐box (also known as *SPL*), *TEOSINTE BRANCHED 1/CYCLOIDEA/PCF* (*TCP*), TGACG‐binding (*TGA*), *ROXY*, and *YABBY* (*YAB*)—also participate in early anther development (Chen et al., 1999; Li, Tian, et al., 2025; Siegfried et al., 1999; Tao et al., 2024; Unte et al., 2003; Xing et al., 2010; Xing & Zachgo, 2008). Unlike the severe defects observed in *spl/nzz* single mutants, most members of these families exhibit substantial functional redundancy. Single mutants typically display few or no detectable developmental abnormalities, whereas higher‐order mutants recapitulate phenotypes comparable to those of *spl/nzz* (Tao et al., 2024; Xing et al., 2010; Yang et al., 1999). Extensive regulatory interactions have been documented among these factors. For example, TCP proteins physically interact with SPL/NZZ to regulate ovule morphogenesis and directly repress transcription of *TGA* and *ROXY* family genes during early anther development (Tao et al., 2024; Wei et al., 2015), highlighting the complexity of the transcriptional regulatory network governing reproductive development.

Among these families, the *SPL* family (formerly known as the SBP‐box family) plays pleiotropic roles in plant growth, development, and responses to environmental stress (Bu et al., 2025; Huijser & Schmid, 2011; Wang et al., 2009). All SPL proteins contain a highly conserved SBP DNA‐binding domain that specifically recognizes the GTAC core cis‐regulatory element (Birkenbihl et al., 2005; Cardon et al., 1999). The Arabidopsis genome encodes 17 SPL proteins, which are phylogenetically divided into two groups. Group I members are not regulated by miR156, whereas most Group II members are post‐transcriptionally repressed by miR156, with *SPL8* representing the only known exception within this group (Xing et al., 2010). Functional studies have shown that *spl8* mutants exhibit partial male sterility in early‐arising flowers, a phenotype that gradually recovers during subsequent floral development. Specifically, *spl8‐1* plants display reduced anther size, fewer anther locules, impaired pollen production, and shortened filaments, indicating that defects in male reproductive organ development underlie the early‐flower sterility phenotype (Unte et al., 2003). Subsequent studies demonstrated that multiple miR156‐targeted *SPL* genes (*SPL2*, *SPL9*, *SPL10*, *SPL11*, *SPL13*, and *SPL15*) also contribute to anther development. Ectopic expression of *MIR156b* in the *spl8‐1* background suppresses these genes and results in complete male sterility, strongly supporting functional redundancy between *SPL8* and the miR156‐targeted *SPL* genes during anther development (Xing et al., 2010). In addition, *SPL8* cooperates with the brassinosteroid signaling effector *BES1‐interacting Myc‐like1* (*BIM1*) to regulate early anther formation (Xing et al., 2013). Despite these advances, the molecular mechanisms by which *SPL8* regulates anther development, particularly its downstream targets and interacting partners, remain largely unknown.

The YABBY and TCP transcription factor families represent two additional regulatory groups involved in plant morphogenesis. The six Arabidopsis *YABBY* genes encode proteins containing an N‐terminal C2C2 zinc finger domain and a conserved C‐terminal YABBY domain. Based on their biological functions, they are classified into vegetative (*FILAMENTOUS FLOWER* (*FIL)/YAB1*, *YAB2*, *YAB3*, and *YAB5*) and reproductive (*INNER NO OUTER* (*INO/YAB4*) and *CRABS CLAW* (*CRC/YAB6*)) subgroups (Bowman & Smyth, 1999; Chen et al., 1999; Sawa et al., 1999; Siegfried et al., 1999; Villanueva et al., 1999). Vegetative *YABBY* genes are predominantly expressed in the abaxial tissue of lateral organs. Although single mutants exhibit few obvious developmental defects, higher‐order mutants display severe abnormalities in leaf polarity and morphology, demonstrating extensive functional redundancy (Sarojam et al., 2010). Notably, mutation of *FIL* results in filamentous floral structures, suggesting an additional role in reproductive development (Chen et al., 1999; Sawa et al., 1999). In contrast, the reproductive *YABBY* genes *INO* and *CRC* specifically regulate the formation of the ovule outer integument and carpel development, respectively (Bowman & Smyth, 1999; Villanueva et al., 1999). However, the roles of *YABBY* genes in early anther development and their crosstalk with other transcription factor families remain largely unexplored.

The TCP family comprises 24 members in Arabidopsis and regulates a wide range of developmental and physiological processes (Manassero et al., 2013; Viola et al., 2023; Viola & Gonzalez, 2023). Similar to the *YABBY* family, *TCP* genes exhibit extensive functional redundancy, with pronounced developmental defects becoming apparent only in higher‐order *tcp* mutants (Wang et al., 2025). Recent studies have uncovered critical roles for miR319‐targeted *TCP* genes during early anther development. *TCP2*, *TCP3*, *TCP4*, *TCP10*, and *TCP24* promote early anther development by activating the expression of *TGA9*, *TGA10*, and *ROXY2* (Tao et al., 2024), whereas *TCP24* additionally regulates anther dehiscence (Wang et al., 2015). Furthermore, *TCP5*, a homolog of the same subfamily, has recently been identified as a potential regulator of early anther development (Li, Tian, et al., 2025), further expanding the functional repertoire of TCP transcription factors in reproductive development. Nevertheless, the complete set of TCP family members involved in anther development, as well as their potential coordination with other transcription factor families, remains to be determined.

Although the individual roles of SPL, YABBY, and TCP transcription factor families in anther development have been increasingly recognized, whether and how these families coordinate to regulate this process through direct interactions remains elusive. Given that SPL/NZZ physically interacts with TCP proteins during ovule development and that *YABBY* genes function in close spatiotemporal coordination during anther morphogenesis (Goldshmidt et al., 2008; Wei et al., 2015), we hypothesized that SPL8 similarly participates in cross‐family regulatory interactions with specific YABBY and TCP proteins to regulate early anther development.

In this study, we identify and characterize a bimodal regulatory pathway underlying SPL8‐dependent anther development. Through biochemical, transcriptional, genetic, and spatiotemporal expression analyses, we define a previously unrecognized regulatory module, the *SPL8*–*YAB3*/*TCP19* axis, in which SPL8 physically interacts with both YAB3 and TCP19 while directly activating their transcription. This regulatory module establishes a hierarchical transcription factor network that promotes anther morphogenesis, thereby providing new mechanistic insights into anther development and identifying potential molecular targets for crop improvement and hybrid breeding.

## RESULTS

### Identification of SPL8‐interacting transcription factors

To systematically identify SPL8‐interacting partners, we performed a yeast two‐hybrid (Y2H) screen using SPL8 as the bait against an Arabidopsis transcription factor (TF) library (Ou et al., 2011). Twelve putative SPL8‐interacting TFs were identified, representing members of the AP2/EREBP, bHLH, HD‐ZIP, HSF, MYB, WRKY, and YABBY families (Table 1).

**Table 1:** Candidate interactors of SPL8 identified via Y2H screening against an Arabidopsis TF library

Gene ID	Family	Description
AT1G49120	AP2‐EREBP	Integrase‐type DNA‐binding super family protein
AT1G53170	AP2‐EREBP	Ethylene response factor 8
AT2G40350	AP2‐EREBP	Integrase‐type DNA‐binding super family protein
AT4G17490	AP2‐EREBP	Ethylene responsive element binding factor 6
AT1G51070	bHLH	basic Helix‐Loop‐Helix 115
AT5G46880	HD‐ZIP	Homeodomain‐leucine zipper class IV/HDG5
AT4G13980	HSF	Heat shock transcription factor A5/HSFA5
AT5G54230	MYB	Myb domain protein 49
AT1G64000	WRKY	WRKY DNA‐binding protein 56
AT3G56400	WRKY	WRKY DNA‐binding protein 70
AT4G01250	WRKY	WRKY DNA‐binding protein 22
AT4G00180	YABBY	YABBY family protein/YAB3

To validate these interactions in planta, bimolecular fluorescence complementation (BiFC) assays were subsequently performed. Among the 12 candidates, six proteins—bHLH115, WRKY22, WRKY56, WRKY70, YAB3, and HSFA5—were confirmed as SPL8 interactors (Figure 1). Notably, SPL8–YAB3 consistently produced abundant BiFC‐positive nuclei per microscopic field.

**Figure 1:**
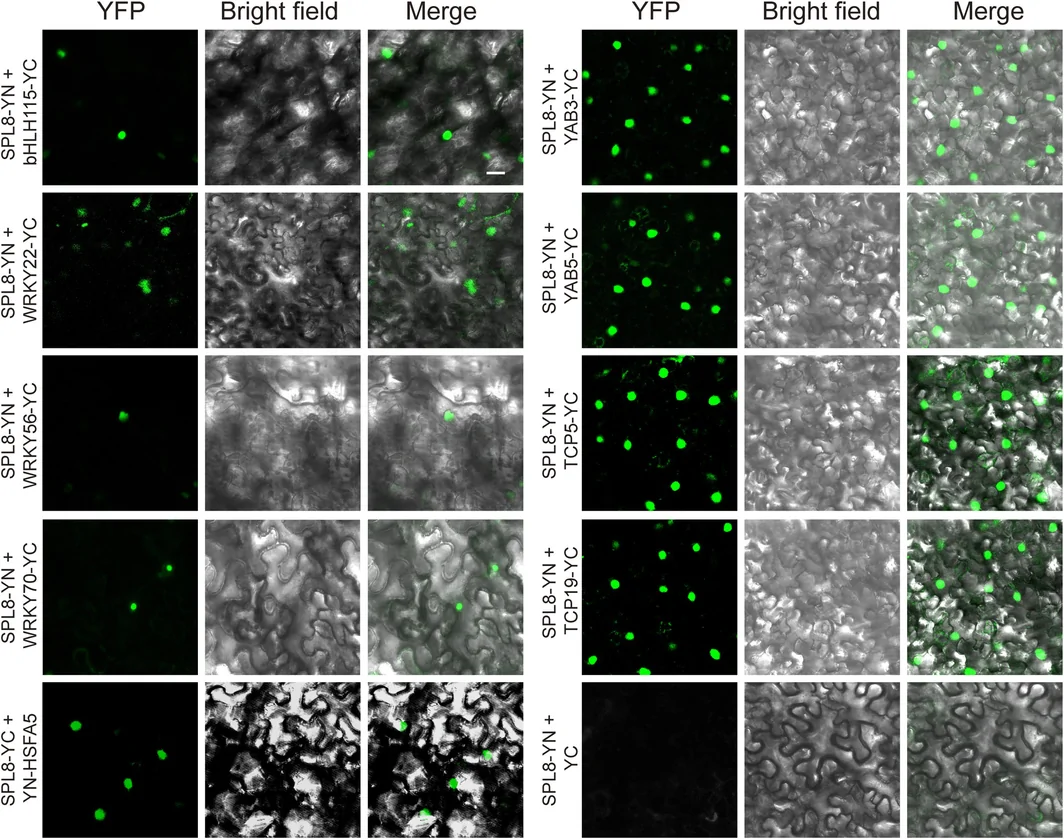
BiFC analysis of interactions between SPL8 and candidate transcription factors. BiFC assays were performed to validate physical interactions between SPL8 and candidate transcription factors. Reconstituted YFP fluorescence indicates protein–protein interactions in *N. benthamiana* nuclei. SPL8‐YN co‐expressed with empty YC served as the negative control. YN and YC represent the N‐ and C‐terminal fragments of YFP, respectively. Scale bar, 20 μm.

We next examined additional members of the YABBY and TCP transcription factor families. BiFC assays confirmed that SPL8 interacted with YAB2, YAB4, and YAB5, but not with YAB1/FIL or YAB6/CRC (Figure 1; Figure S1). Similar to the SPL8–YAB3 interaction, the SPL8–YAB5 interaction also generated abundant BiFC‐positive nuclei. Moreover, SPL8 interacted with multiple TCP proteins, including TCP5, TCP7, TCP8, TCP9, TCP13, TCP15, TCP17, TCP19, TCP20, and TCP23 (Figure 1; Figure S1). Among these interactions, SPL8–TCP5 and SPL8–TCP19 produced BiFC‐positive nuclei comparable in number to those observed for SPL8‐YAB3 and SPL8‐YAB5 (Figure 1).

We further examined interactions between SPL8 and several key regulators involved in floral and anther development. SPL8 interacted with APETALA1 (AP1) and CAULIFLOWER (CAL), but no interaction was detected with AGAMOUS (AG), PISTILLATA (PI), SPL/NZZ, ROXY1, ROXY20, or TOPLESS (TPL) (Figure S1; Tables S1 and S2). In addition, SPL8 formed homodimers and heterodimers with SPL3, SPL4, SPL5, and SPL9 (Figure S1).

Given the abundant BiFC signals, we focused on YAB3, YAB5, TCP5, and TCP19 for their mutual interactions. All pairwise combinations among these four proteins—including YAB3–YAB5, YAB3–TCP5, YAB3–TCP19, YAB5–TCP5, YAB5–TCP19, and TCP5–TCP19—yielded positive signals (Figure S1). Additionally, SPL9, a functional homolog of SPL8 implicated in anther development, also interacted with YAB3 and TCP19. Both YAB3 and TCP19 also interacted with TPL (Figure S1).

Collectively, 26 TFs were confirmed as SPL8‐interacting proteins by BiFC analysis (Table S1). Among these candidates, YAB3 and TCP19 were selected for further functional characterization.

### Validation of pairwise interactions among SPL8, YAB3, and TCP19

To further validate the interaction network among SPL8, YAB3, and TCP19, Y2H and fluorescence resonance energy‐transfer–fluorescence lifetime imaging microscopy (FRET‐FLIM) assays were performed. Y2H assays confirmed interactions among all three protein pairs: SPL8–YAB3, SPL8–TCP19, and YAB3–TCP19. However, these interactions were weaker than the positive control interaction. Yeast transformants grew normally on triple‐dropout (TDO) medium but failed to survive on stringent quadruple‐dropout (QDO) selective medium, indicating relatively weak or moderate interactions in the yeast system (Figure 2A).

**Figure 2:**
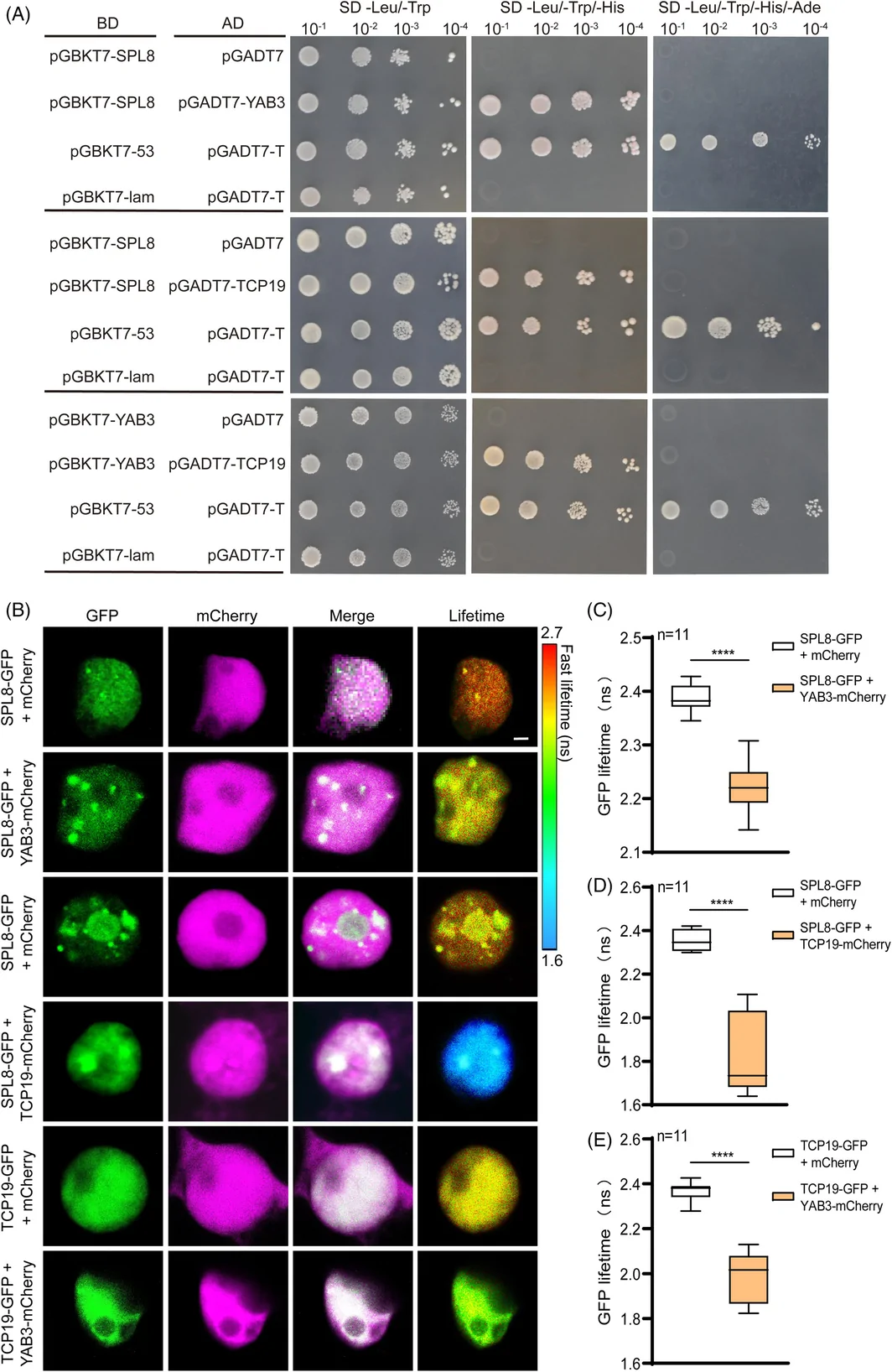
Validation of physical interactions among SPL8, YAB3, and TCP19 by yeast two‐hybrid (Y2H) and FRET‐FLIM assays. (A) Y2H assays showing pairwise interactions among SPL8, YAB3, and TCP19. Different combinations of bait and prey constructs were transformed into Y2H Gold yeast cells and grown on selective media: DDO (SD/‐Leu/‐Trp), TDO (SD/‐Leu/‐Trp/‐His), and QDO (SD/‐Leu/‐Trp/‐His/‐Ade). The pGBKT7‐53/pGADT7‐T combination served as the positive control, whereas pGBKT7‐Lam/pGADT7‐T served as the negative control. (B) FRET‐FLIM analysis of interactions among SPL8, YAB3, and TCP19 in *N. benthamiana* nuclei. GFP was used as the donor fluorophore and mCherry as the acceptor fluorophore, with excitation wavelengths set at 488 and 561 nm, respectively. Fluorescence lifetimes of the two fluorophores were acquired and quantified. SPL8‐GFP co‐expressed with free mCherry, and TCP19‐GFP co‐expressed with free mCherry were included as negative controls. The color bar indicates fluorescence lifetime (nanoseconds). Scale bar, 2 μm. (C–E) Quantitative analysis of fluorescence lifetimes for the indicated protein combinations. (C) GFP fluorescence lifetimes (ns) of SPL8‐GFP co‐expressed with free mCherry (control) or YAB3‐mCherry. (D) GFP fluorescence lifetimes (ns) of SPL8‐GFP co‐expressed with free mCherry (control) or TCP19‐mCherry. (E) GFP fluorescence lifetimes (ns) of TCP19‐GFP co‐expressed with free mCherry (control) or YAB3‐mCherry. Reduced GFP lifetime indicates FRET resulting from protein–protein interaction. Boxplots depict the negative control and test combinations; center lines represent the medians, box bounds denote the 25th and 75th percentiles, and whiskers indicate the minimum and maximum values. Data were obtained from three independent biological replicates (*n* = 11 per group). Statistical significance was determined by unpaired *t*‐test with Welch's correction (****P < 0.0001).

We next examined these interactions in plant cells using FRET‐FLIM assays in *Nicotiana benthamiana* leaves. Co‐expression of SPL8‐GFP with either YAB3‐mCherry or TCP19‐mCherry resulted in nuclear co‐localization of the two proteins (Figure 2B). Compared with the SPL8‐GFP/empty‐mCherry control, co‐expression with YAB3‐mCherry or TCP19‐mCherry caused a significant reduction in the fluorescence lifetime of SPL8‐GFP (Figure 2C,D), indicating energy transfer between the donor and acceptor fluorophores. Similarly, the fluorescence lifetime of TCP19‐GFP was significantly reduced in the presence of YAB3‐mCherry (Figure 2E), verifying the YAB3–TCP19 interaction in planta.

Collectively, the Y2H and FRET‐FLIM results demonstrate pairwise interactions among SPL8, YAB3, and TCP19 in both yeast and plant cells. Together with the BiFC data, these findings support the existence of a nuclear‐localized protein–protein interaction module involving these three proteins, prompting us to examine whether *SPL8* transcriptionally regulates *YAB3* and *TCP19*.

### *SPL8* directly activates the transcription of *YAB3* and *TCP19*

A dexamethasone/glucocorticoid receptor (DEX/GR)‐inducible *35S:SPL8‐GR* system was used to determine whether *SPL8* directly regulates the expression of *YAB3* and *TCP19*.

RT‐qPCR analysis showed that DEX‐induced activation of *SPL8* significantly increased the transcript levels of *YAB3* and *TCP19*. Importantly, induction of both genes was maintained in the presence of cycloheximide (CHX), a potent inhibitor of de novo protein synthesis. These results indicate that *SPL8*‐mediated activation of *YAB3* and *TCP19* does not require newly synthesized intermediary proteins, suggesting that both genes are direct transcriptional targets of *SPL8* (Figure 3A,B).

**Figure 3:**
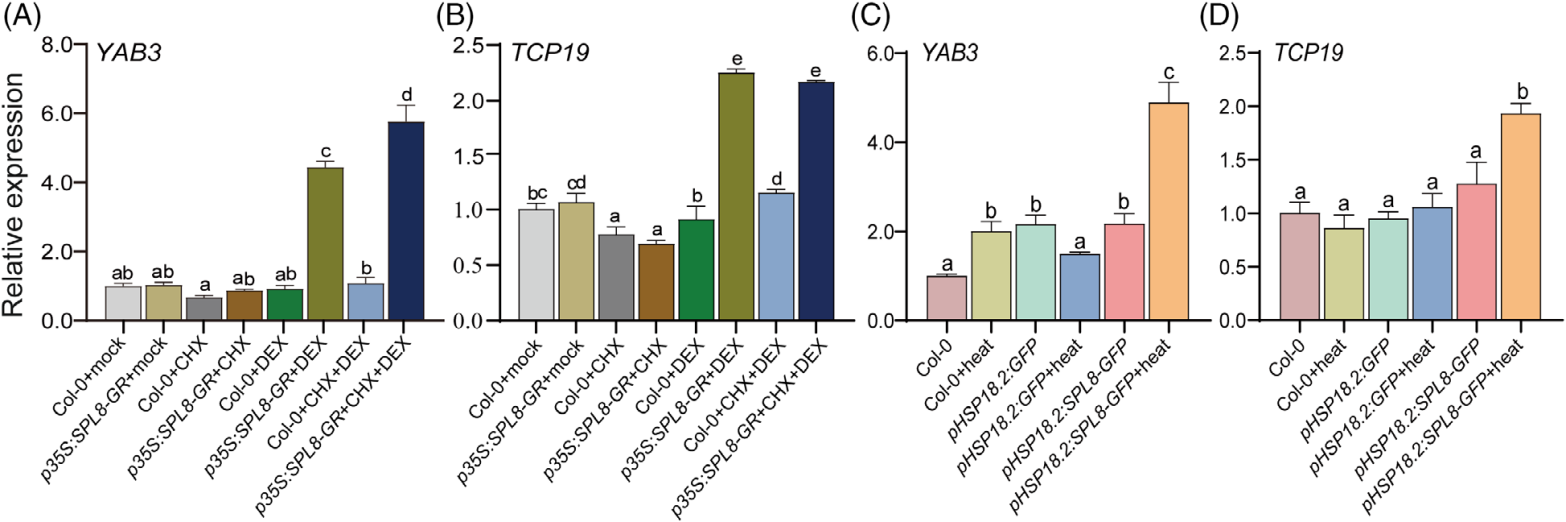
*SPL8* activates *YAB3* and *TCP19* expression through inducible expression systems. (A, B) RT‐qPCR analysis of *YAB3* and *TCP19* expression in 2‐week‐old Col‐0 and *35S:SPL8‐GR* seedlings treated with mock (0.1% ethanol), DEX, CHX, or DEX + CHX. (C, D) RT‐qPCR analysis of *SPL8*‐dependent activation of *YAB3* and *TCP19* using the heat‐inducible system. Two‐week‐old Col‐0, *pHSP18.2:GFP*, and *pHSP18.2:SPL8‐GFP* seedlings were subjected to heat treatment at 37°C for 2 h, followed by recovery at 22–24°C for 6 h. Data are shown as mean ± SE from three biological replicates. Different letters indicate significant differences determined by one‐way anova followed by Tukey's multiple comparison test (*P* < 0.05).

To further validate this regulatory relationship, we generated transgenic plants using an independent heat‐inducible system driven by the *HSP18.2* promoter (Takahashi et al., 1992). First, β‐glucuronidase (GUS) staining of *pHSP18.2:GUS* reporter plants demonstrated strong and sustained heat‐induced promoter activity in seedlings and inflorescences at 2, 4, 8, and 24 h after heat treatment (Figure S2c–i). We subsequently examined *pHSP18.2:SPL8‐GFP* transgenic lines, with *pHSP18.2:GFP* as the control. After a 2‐h heat shock, SPL8‐GFP protein exclusively accumulated in the nucleus (Figure S2j–l).

Consistent with the DEX/GR induction system, heat‐induced expression of *SPL8* significantly increased the transcript levels of *YAB3* and *TCP19* compared with the *pHSP18.2:GFP* control plants (Figure 3C,D). Together, these results demonstrate that *SPL8* positively regulates the expression of *YAB3* and *TCP19* and suggest that both genes function as direct downstream targets of *SPL8*.

### SPL8 physically associates with the regulatory regions of *YAB3* and *TCP19*

To determine whether SPL8 associates with the *YAB3* and *TCP19* loci *in vivo*, we performed chromatin immunoprecipitation sequencing (ChIP‐seq) on *pHSP18.2:SPL8‐GFP* transgenic seedlings following heat induction. We identified 7094 putative SPL8‐binding peaks (Table S3). Among these, significant enrichment was detected at the regulatory regions of *YAB3* and *TCP19* (Figure 4A,C).

**Figure 4:**
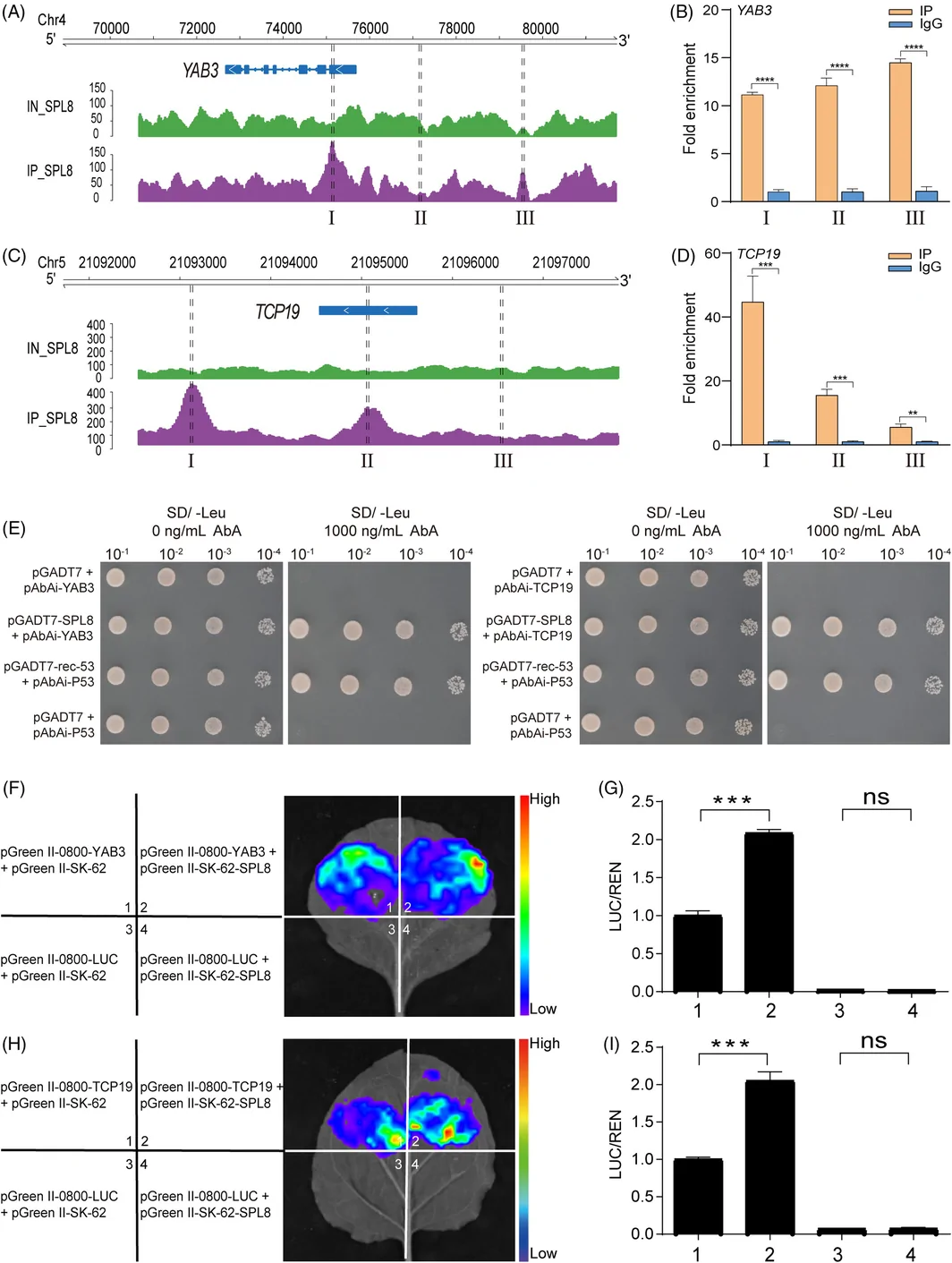
SPL8 directly binds to and activates *YAB3* and *TCP19*. (A, C) Genome browser views showing SPL8‐binding peaks at the *YAB3* and *TCP19* genomic loci identified by ChIP‐seq. Regions I–III indicate the fragments selected for subsequent ChIP‐qPCR validation. (B, D) ChIP‐qPCR validation of SPL8 enrichment at the indicated regions of *YAB3* and *TCP19*. IgG served as the negative control. Data are presented as mean ± SE from three biological replicates. Significant differences are indicated as ** (*P* < 0.01), *** (*P* < 0.001), **** (*P* < 0.0001). (E) Yeast one‐hybrid (Y1H) assays demonstrating direct binding of SPL8 to the *YAB3* distal promoter (−4804 to −3596 bp) and the *TCP19* coding region (+101 to +848 bp). Bait constructs containing the target sequences were integrated into Y1HGold cells and subsequently transformed with the SPL8 prey construct. Yeast cells were grown on SD/‐Leu and SD/‐Leu/AbA media. The combination of pGADT7‐rec‐53 and pAbAi‐P53 served as the positive control, whereas empty pGADT7 and pAbAi‐P53 served as the negative control. (F–I) Dual‐luciferase assays showing that SPL8 activates the transcription of *YAB3* and *TCP19*. (F, H) Representative luminescence images of *N. benthamiana* leaves co‐transformed with the indicated effector and reporter constructs; the color scale represents luminescence intensity. (G, I) Quantification of relative luciferase activity. Statistical differences are marked as ns (not significant) or *** (*P* < 0.001).

Based on the distribution of SPL8‐binding peaks, three genomic regions from each locus were selected for ChIP‐qPCR validation (Figure 4A,C; Table S4). Consistent with the ChIP‐seq results, strong SPL8 enrichment was detected at two major peak regions in *YAB3* (Regions I and III) and *TCP19* (Regions I and II). Of note, significant enrichment was also detected at *YAB3* Region II and *TCP19* Region III, although these regions fell outside the peaks in the genome‐wide analysis (Figure 4A–D).

To further confirm the DNA‐binding activity of SPL8, yeast one‐hybrid (Y1H) and dual‐luciferase reporter assays were performed using a 1208 bp distal promoter fragment of *YAB3* harboring Region III and a 747 bp *TCP19* coding‐region fragment harboring Region II (Figure S3a). Both fragments contained multiple copies of the canonical SPL‐binding motif GTAC (Figure S3b,c). In the Y1H assay, yeast cells expressing SPL8 together with either the *YAB3* promoter fragment or the *TCP19* genomic fragment exhibited robust growth on selective medium, demonstrating that SPL8 can bind these DNA regions directly (Figure 4E).

We next examined whether SPL8 regulates the transcriptional activity of these regions using dual‐luciferase reporter assays in *N. benthamiana* leaves. Compared with the empty vector control, SPL8 co‐expression significantly enhanced luciferase activity driven by the *YAB3* promoter fragment and the *TCP19* reporter fragment (Figure S4; Figure 4F–I). These results indicate that SPL8 directly associates with regulatory regions of *YAB3* and *TCP19* and promotes their transcriptional activation.

Together with the protein–protein interaction data, these findings establish the *SPL8*–*YAB3*/*TCP19* regulatory module, in which *SPL8* directly promotes the transcription of *YAB3* and *TCP19* in addition to interacting with their encoded proteins. These results prompted further investigation of the spatiotemporal expression patterns and functional roles of this module during anther development.

### Overlapping spatiotemporal expression of *SPL8* , *YAB3* , and *TCP19* in early developing anthers

To investigate the potential functional relationship among *SPL8*, *YAB3*, and *TCP19*, we characterized their tissue‐specific and spatial expression patterns using RT‐qPCR, GUS staining, GFP fluorescence analysis, and RNA *in situ* hybridization.

Tissue‐specific RT‐qPCR analysis revealed distinct but partially overlapping expression patterns among the three genes. *SPL8* transcripts were predominantly detected in inflorescences, moderately expressed in maturing siliques, and present at very low levels in seedlings (Figure 5A). In contrast, *YAB3* exhibited a broader expression pattern, with relatively high transcript abundance in seedlings, leaves, inflorescences, and mature siliques (Figure 5B). *TCP19* expression was enriched mainly in inflorescences and maturing siliques, while relatively low expression was detected in other vegetative tissues (Figure 5C).

**Figure 5:**
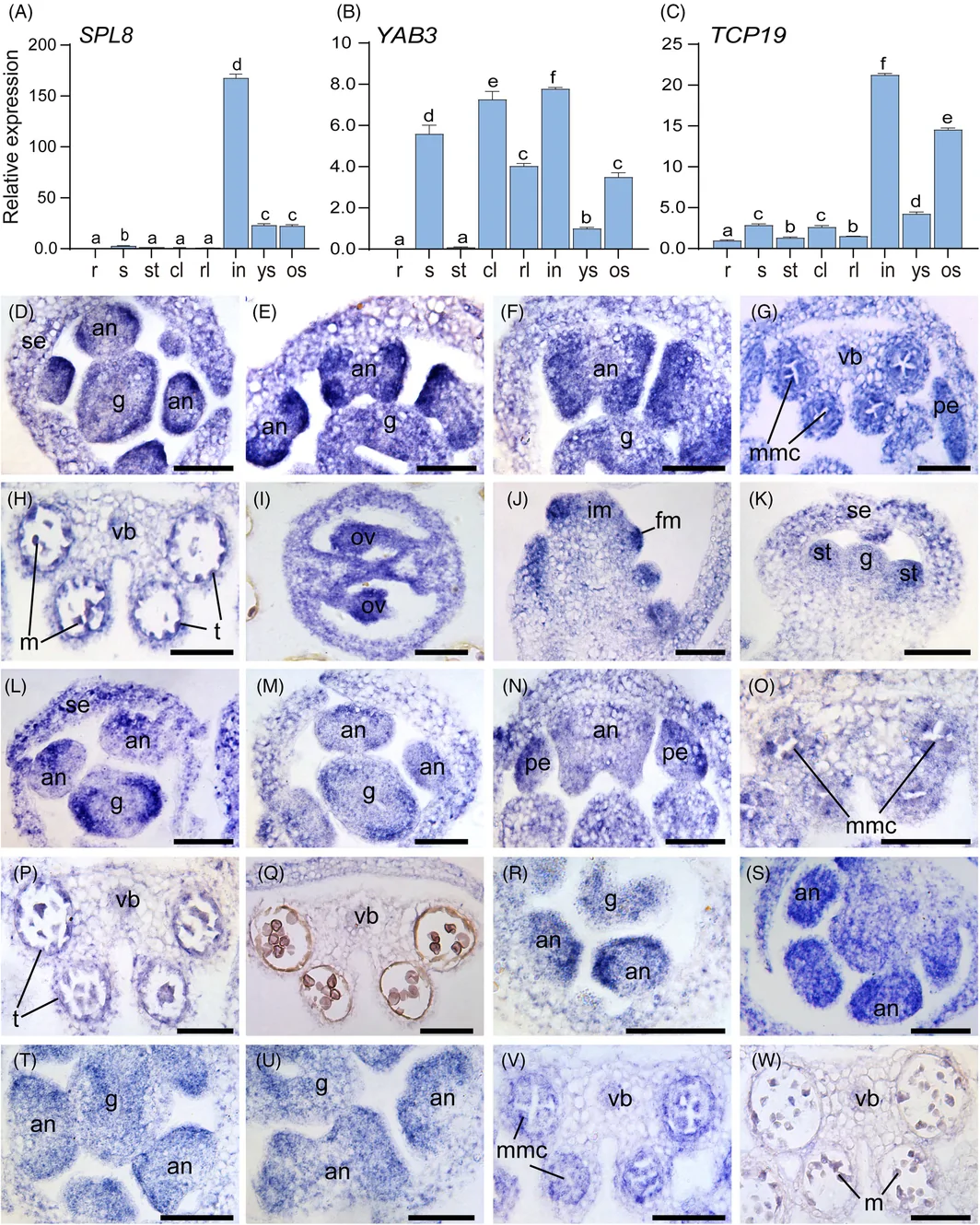
Expression patterns of *SPL8*, *YAB3*, and *TCP19* in different tissues revealed by RT‐qPCR and RNA *in situ* hybridization. (A–C) RT‐qPCR analysis of *SPL8*, *YAB3*, and *TCP19* expression in different tissues. Data are presented as mean ± SE (*n* = 3). Different letters indicate statistically significant differences at *P* < 0.05. (D–W) RNA *in situ* hybridization showing the spatial expression patterns of *SPL8* (D–I), *YAB3* (J–Q), and *TCP19* (R–W) in developing flowers and anthers. *SPL8* transcripts accumulated strongly in early anthers (D–G), with weaker signals detected in the tapetum, microspores, and vascular bundle at post‐meiotic stages (H). Strong *SPL8* expression was also observed in developing petals and ovules (G, I). *YAB3* transcripts were abundant in the inflorescence and floral primordia (J) and were detected throughout developing floral organs (K–N). During anther development, however, *YAB3* expression declined markedly during meiosis (O, P) and was nearly undetectable at late developmental stages (Q). *TCP19* displayed an expression pattern largely similar to that of *SPL8*, with strong expression in early anthers (R–U), reduced transcript accumulation during meiosis (V), and weak or undetectable expression in post‐meiotic anthers (W). (A–C) cl, cauline leaf; in, inflorescence; os, old silique; r, root; rl, rosette leaf; s, seedling; st, stem; ys, young silique. (D–W) an, anther; fm, floral meristem; g, gynoecium; im, inflorescence meristem; m, microspore; mmc, microspore mother cell; ov, ovule; pe, petal; se, sepal; st, stamen; t, tapetum; vb, vascular bundle. Scale bars, 50 μm.

Consistent with the RT‐qPCR results, GUS assays of *pSPL8:GUS*, *pYAB3:GUS*, and *pTCP19:GUS* reporter lines revealed strong signals in developing anthers, with weaker signals detected in gynoecia (Figure S5a–c). Similarly, GFP fluorescence in *pSPL8:SPL8‐GFP*, *pYAB3:YAB3‐GFP*, and *pTCP19:TCP19‐GFP* transgenic lines was observed in developing anthers and gynoecia. All three fusion proteins were predominantly localized to the nucleus, consistent with their roles as transcription factors (Figure S5d–f).

RNA *in situ* hybridization further defined the spatial distribution of these transcripts during floral development. *SPL8* expression was detected strongly in young anthers prior to meiosis, particularly in microspore mother cells (Figure 5D–G). Following meiosis, weaker *SPL8* signals remained detectable in microspores, tapetal cells, and anther vascular tissues (Figure 5H). In addition, *SPL8* transcripts were detected in developing petals and ovules (Figure 5G,I). *YAB3* transcripts were initially abundant in floral meristems (Figure 5J) and subsequently detected throughout developing floral organs. In developing anthers and petals, *YAB3* expression was enriched preferentially in abaxial regions (Figure 5K–N). During and after meiosis, *YAB3* expression gradually decreased in microspore mother cells, tapetal cells, and vascular tissues, becoming undetectable at late anther stages (Figure 5O–Q). *TCP19* showed relatively uniform expression in young anthers and gynoecia (Figure 5R–U). Similar to *YAB3*, *TCP19* expression decreased during meiosis and was not detectable in mature anthers (Figure 5V, W).

Together, these results demonstrate that *SPL8*, *YAB3*, and *TCP19* exhibit overlapping expression domains during early anther development, particularly in tapetum, microspore mother cells, and vascular bundles. Moreover, their encoded proteins accumulate predominantly in the nucleus. These overlapping spatial and temporal expression patterns support a potential coordinated role for the *SPL8*–*YAB3*/*TCP19* module during early anther development.

### Genetic evidence for the essential roles of *SPL8* , *YAB3* , and *TCP19* in anther development

To investigate the biological functions of the *SPL8*–*YAB3*/*TCP19* module, we generated and characterized a series of genetic materials. We first examined the *yab3* T‐DNA insertion mutant, a CRISPR/Cas9‐generated *tcp19* mutant, and the previously characterized *spl8‐1* mutant (Unte et al., 2003). RT‐PCR analysis confirmed that all three mutants represented null alleles, exhibiting complete loss of gene expression (Figure S6). Phenotypic analysis showed that *yab3* and *tcp19* single mutants developed normally and exhibited no obvious anther defects, suggesting substantial functional redundancy within the YABBY and TCP families.

To determine whether *YAB3* and *TCP19* contribute to the *SPL8*‐dependent anther phenotype, we generated the *spl8‐1 yab3* and *spl8‐1 tcp19* double mutants, as well as the *spl8‐1 yab3 tcp19* triple mutant. All higher‐order mutants displayed phenotypes comparable to that of the *spl8‐1* single mutant, including partial male sterility predominantly affecting early‐emerging flowers, with no evident enhancement of the phenotype. The absence of synergistic defects suggests that additional YABBY and TCP family members may compensate for the loss of *YAB3* and *TCP19* during anther development.

To address potential functional redundancy, we generated both constitutive overexpression lines and SRDX‐mediated dominant repression lines (Table 2). For overexpression analysis, the constitutive *UBQ10* promoter was used to drive ubiquitous expression. *pUBQ10:YAB3‐3HA* plants exhibited severe floral abnormalities, including increased stamen number and suppressed gynoecium development, indicative of a homeotic conversion of female reproductive organs into stamens (Figure S7a1–d3). In contrast, *pUBQ10:TCP19‐3HA* plants showed no obvious developmental defects. Despite multiple independent transformation attempts, we were unable to recover any *pUBQ10:SPL8‐3HA* transgenic plants.

**Table 2:** List of constructs and phenotypes of the transgenic plants^a^

Constructs	Major phenotypes
pUBQ10:YAB3‐3HA	Extra stamens; pistil‐to‐stamen conversion
pUBQ10:TCP19‐3HA	No phenotypic changes
pUBQ10:YAB3‐SRDX	No phenotypic changes
pUBQ10:TCP19‐SRDX	Stamen feminization; abnormal gynoecium
pYAB3:YAB3‐SRDX	Petal filamentation and stamen clavation
pTCP19:3HA‐TCP19‐SRDX	Stamen feminization; abnormal gynoecium
pTCP19:TCP19‐SRDX	Stamen feminization; abnormal gynoecium
pSPL8:SPL8‐SRDX	Defective anthers producing no pollen, short filaments
pSPL8:YAB3‐SRDX	Anther clavation and filamentation
pSPL8:TCP19‐SRDX	Defective anthers producing no pollen
pDRL1:SPL8‐SRDX	Defective anthers producing no pollen
pDRL1:YAB3‐SRDX	Defective anthers producing no pollen
pDRL1:TCP19‐SRDX	Defective anthers producing no pollen
pREM22:YAB3‐SRDX	Two pollen sacs per anther with no pollen
pREM22:TCP19‐SRDX	Stamen feminization; abnormal gynoecium
pA9:YAB3‐SRDX	Defective anthers, reduced pollen production
pA9:TCP19‐SRDX	Defective anthers, reduced pollen production

^a^ *DRL1*, *dihydroflavonol 4‐reductase‐like1* (Tang et al., 2009); *REM22*, *reproductive meristem22* (Romanel et al., 2011).

To investigate the effects of transcriptional repression, *SPL8*‐, *YAB3*‐, and *TCP19‐SRDX* fusion constructs were expressed under the control of promoters with distinct spatial expression patterns, including the constitutive *UBQ10* promoter, their respective endogenous promoters, the tapetum‐specific *A9* promoter, the anther‐specific *DRL1* promoter, and the early anther‐specific *REM22* promoter (Paul et al., 1992; Romanel et al., 2011; Tang et al., 2009) (Figure S2a,b; Table 2). *SPL8‐SRDX* lines driven by the native *SPL8* promoter (*pSPL8*) or the *DRL1* promoter (*pDRL1*) were included as reference lines, based on the established role of *SPL8* in anther development (Unte et al., 2003; Xing et al., 2010). *pSPL8:SPL8‐SRDX* plants exhibited short stamens lacking mature pollen (Figure 6A–F), whereas *pDRL1:SPL8‐SRDX* plants displayed severe anther defects accompanied by complete pollen abortion (Figure 6M–O). Both phenotypes were more severe than those observed in the *spl8* mutant, consistent with the dominant‐negative effect of SRDX‐mediated transcriptional repression.

**Figure 6:**
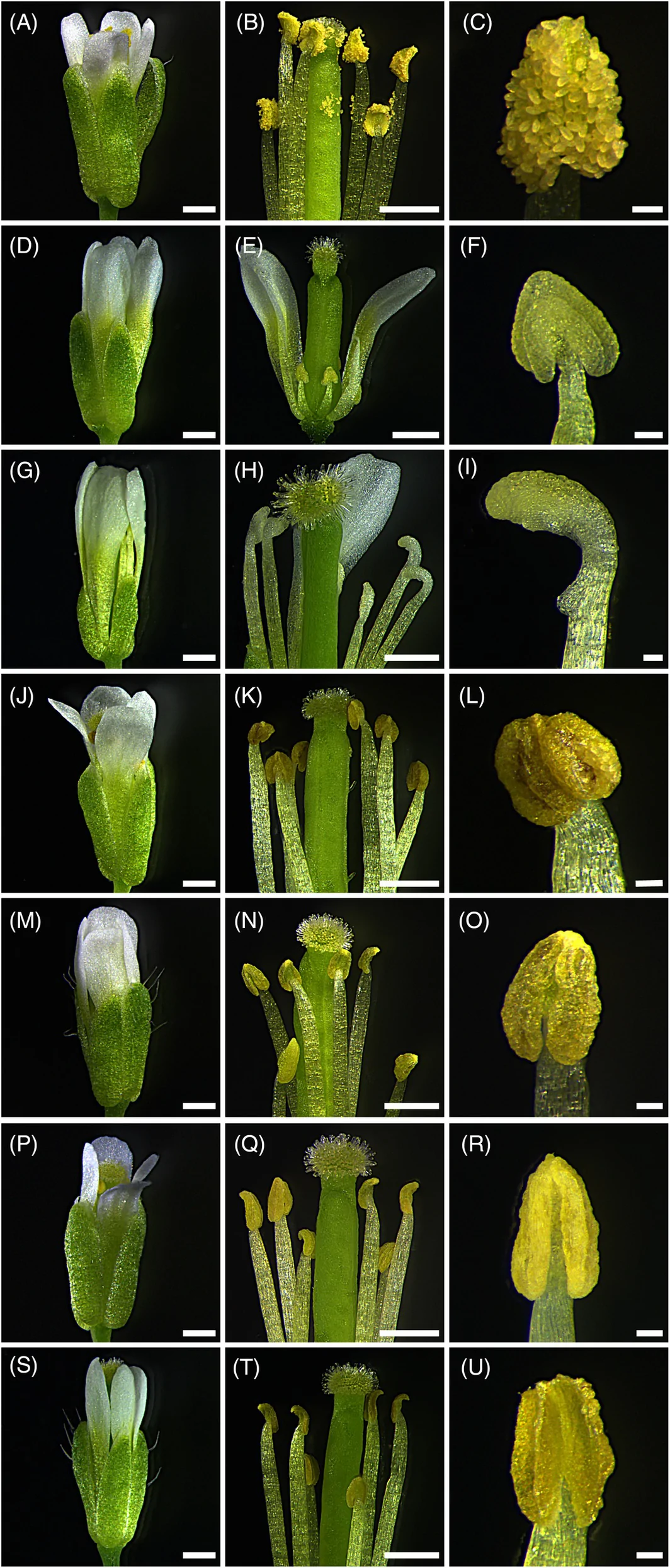
Phenotypic characterization of *SPL8‐*, *YAB3‐*, and *TCP19‐SRDX* repression lines driven by *SPL8* and *DRL1* promoters. (A–C) Wild‐type Col‐0. (D–F) *pSPL8:SPL8‐SRDX*; (G–I) *pSPL8:YAB3‐SRDX*; (J–L) *pSPL8:TCP19‐SRDX*. (M–O) *pDRL1:SPL8‐SRDX*; (P–R) *pDRL1:YAB3‐SRDX*; (S–U) *pDRL1:TCP19‐SRDX*. For each genotype, images show intact flower, flower with perianth removed, and isolated anther. Scale bars, 50 μm for (C, F, I, L, O, R, U) and 500 μm for all other panels.

Given that the roles of *YAB3* and *TCP19* in anther development remain largely uncharacterized, we performed a comprehensive promoter survey using SRDX‐mediated repression. *YAB3‐SRDX* exhibited striking promoter‐dependent effects. Expression driven by *pYAB3* resulted in narrow sepals, filamentous petals, and clavate anthers (Figure S7e1–f3), whereas constitutive expression under *pUBQ10* produced no detectable floral defects (Table 2), suggesting that *YAB3* function is highly tissue dependent. Notably, *pSPL8:YAB3‐SRDX* also produced clavate anthers (Figure 6G–I), closely phenocopying the anther phenotype of *pYAB3:YAB3‐SRDX*. Furthermore, both *pDRL1:YAB3‐SRDX* and *pA9:YAB3‐SRDX* caused severe anther defects accompanied by complete pollen abortion (Figure 6P–R; Figure S7g1–g3), closely resembling the phenotype of *pDRL1:SPL8‐SRDX*.

*TCP19‐SRDX* likewise produced promoter‐dependent phenotypes. Constitutive expression driven by *pUBQ10* caused stamen feminization and impaired gynoecium development (Figure S7h1–i3), whereas expression from *pTCP19* (Figure S7j1–j3) or the early anther promoter *pREM22* (Figure S7k1–k6) resulted in similar but less severe defects. Under *pSPL8*, *TCP19‐SRDX* induced moderate anther abnormalities and occasional ectopic hairs on the gynoecium (Figure 6J–L), partially overlapping with the *pDRL1:SPL8‐SRDX* phenotype. Consistent with the effects of *SPL8‐SRDX* and *YAB3‐SRDX*, *pDRL1:TCP19‐SRDX* caused severe anther‐specific defects accompanied by complete pollen abortion (Figure 6S–U).

Collectively, these promoter‐dependent phenotypes, together with the physical and transcriptional interactions described above, support the conclusion that *SPL8*, *YAB3*, and *TCP19* function as a coordinated regulatory module during anther development. Although repression of each gene produced distinct outcomes depending on the promoter used, their remarkably similar phenotypes under *pDRL1*, particularly the shared severe anther defects and complete pollen abortion, prompted us to investigate whether these morphological similarities arose from common or distinct underlying cellular defects.

### Anther section analysis of various promoter‐driven SRDX‐fusion repressor lines

To further characterize the cellular defects caused by repression of SPL8, YAB3, and TCP19, we performed histological analyses of transverse anther sections from the corresponding SRDX fusion lines. We initially focused on the *pDRL1*‐driven repression lines because the *DRL1* promoter confers anther‐specific expression in the tapetum, tetrads, and developing microspores from meiosis onward (Tang et al., 2009). Compared with wild‐type plants, early anther development and meiotic progression (stages 5–7) appeared largely normal in *pDRL1:SPL8‐SRDX* lines (Figure 7A,E; Figure S8a1–a3,b1–b3). However, after meiosis, microspores failed to separate properly from tetrads and remained aggregated in clusters (Figure 7B,F; Figure S8a4,a5,b4,b5). At later stages, the locules were filled with highly vacuolated microspores (Figure 7C,G; Figure S8a6,b6), which subsequently degenerated, leaving the locules largely empty by anthesis (Figure 7D,H; Figure S8a7,a8,b7,b8).

**Figure 7:**
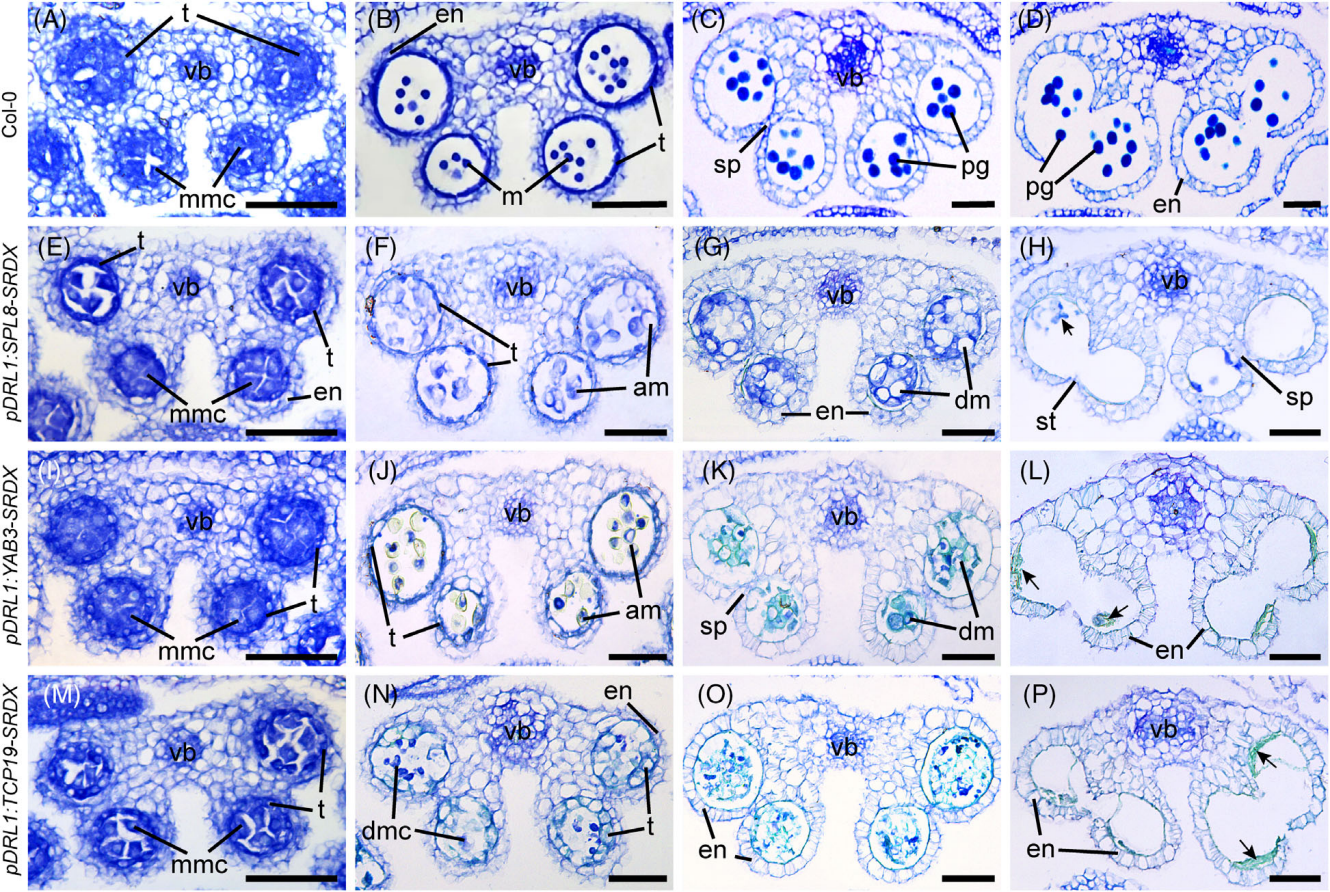
Histological analysis of anther development from meiosis to anthesis in wild‐type and *DRL1*‐driven SRDX chimeric repressor lines. (A–D) Wild‐type Col‐0. Normal anther development from meiosis to pollen maturation. At stage 5–6 (A), microspore mother cells are located in the center of the locules, surrounded by the tapetum and other anther wall layers. At stage 8–9 (B), tetrads and developing microspores are formed, while the tapetum undergoes normal differentiation. At stage 11–12 (C), mature pollen grains accumulate as the tapetum degenerates. By stage 13 (D), the anthers dehisce, and mature pollen is released. (E–H) *pDRL1:SPL8‐SRDX* transgenic line. Microspore mother cells develop normally at stage 5–6 (E). After meiosis, microspores remain enclosed within tetrads and fail to separate properly (F, stage 9–10). By stage 11 (G), the locules are filled with highly vacuolated microspores that progressively degenerate, leaving the locules largely empty by anthesis (H, stage 13). (I–L) *pDRL1:YAB3‐SRDX* transgenic line. Microspore mother cells appear normal at stage 5–6 (I). Following meiosis, however, the tapetum exhibits precocious vacuolation, and microspores develop abnormally (J, stage 9–10). Subsequently, both the tapetum and microspores degenerate (K, stage 11–12), resulting in nearly empty locules by anthesis (L, stage 13). (M–P) *pDRL1:TCP19‐SRDX* transgenic line. Microspore mother cells exhibit normal morphology at stage 5–6 (M). However, they fail to complete meiosis. Concomitantly, the tapetum becomes enlarged and vacuolated, and the meiocytes subsequently degenerate (N, stage 9). Cellular debris derived from the degenerating meiocytes accumulates within the locules at stage 11 (O), leaving the locules almost empty by anthesis (P, stage 13). am, abnormal microspore; dm, degenerated microspore; dmc, degenerating meiotic cell; en, endothecium; m, microspore; mmc, microspore mother cell; pg, pollen grain; sp, septum; st, stomium; t, tapetum; vb, vascular bundle. Arrows indicate degenerative remnants. Scale bars, 50 μm.

By contrast, the *pDRL1:YAB3‐SRDX* and *pDRL1:TCP19‐SRDX* lines exhibited distinct and more severe developmental abnormalities. In both lines, vacuolated tapetal cells surrounding microspore mother cells or meiotic cells were already evident at stages 5–6 (Figure 7I,M; Figure S8c1,c2,d1,d2). Specifically, in *pDRL1:YAB3‐SRDX* anthers, microspores displayed cytoplasmic shrinkage and abnormal development, accompanied by tapetal vacuolation (Figure 7J; Figure S8c3–c5). At later stages, both microspores and the tapetum underwent degeneration, resulting in nearly empty locules by anthesis (Figure 7K,L; Figure S8c6–c8). In *pDRL1:TCP19‐SRDX* anthers, tapetal cells became enlarged and highly vacuolated, and meiotic progression was disrupted. Ultimately, no mature pollen grains were produced, and the locules remained empty at anthesis (Figure 7N–P; Figure S8d3–d8).

To determine whether *YAB3* and *TCP19* also function during earlier stages of anther development, we next examined SRDX repression lines driven by the *REM22* promoter, which has been reported to be active in archesporial cells as well as in the tapetal and middle‐layer cells (Romanel et al., 2011). Transgenic *pREM22:YAB3‐SRDX* plants exhibited a unique early developmental defect in which each anther produced only two pollen sacs instead of the normal four (Figure S9a–k). Despite the reduced number of pollen sacs, microspore mother cells underwent meiosis; however, the tapetum became vacuolated during meiosis (Figure S9h,i) and subsequently degenerated precociously. As a result, developing microspores progressively degenerated, leading to nearly empty anther locules at later stages (Figure S9j,k). This phenotype was largely similar to that observed in *pDRL1:YAB3‐SRDX* lines (Figure 7I–L; Figure S8c1–c8), except for the additional reduction in pollen sac number.

In *pREM22:TCP19‐SRDX* anthers, early development at stage 3 appeared normal (Figure S9l). However, by stage 5, vacuolated tapetal cells were observed surrounding microspore mother cells (Figure S9m). Subsequently, the tapetum became enlarged, with cells exhibiting either dense cytoplasmic contents or extensive vacuolation. Meanwhile, microspore mother cells failed to complete meiosis and progressively degenerated together with the hypertrophied tapetum, ultimately preventing normal pollen formation at later stages (Figure S9n–q). Consistent with other *TCP19‐SRDX* lines, *pREM22:TCP19‐SRDX* plants frequently exhibited feminized anthers and occasionally showed reduced pollen sac numbers (Figure S9r–u).

Collectively, histological analyses demonstrate that although *pDRL1*‐driven *SPL8‐*, *YAB3‐*, and *TCP19‐SRDX* lines exhibit similar macroscopic phenotypes, they display markedly distinct cellular defects. SPL8 repression primarily affects microspore release from tetrads, whereas YAB3 repression causes cytoplasmic shrinkage and tapetal vacuolation, and TCP19 repression results in meiotic arrest and tapetal hypertrophy. These differential cellular effects were further supported by analyses of the *pREM22*‐driven lines, in which repression of YAB3 or TCP19 in early anther tissues disrupted microspore development, phenocopying the defects observed in the corresponding *pDRL1* lines, with the additional reduction of pollen sac number in these *pREM22*‐driven lines. Altogether, these results reveal that the *SPL8–YAB3/TCP19* module integrates transcriptional regulation and protein interactions to coordinate multiple successive steps during anther development, thereby ensuring the production of viable pollen.

## DISCUSSION

### SPL8 physically interacts with multiple YABBY, TCP, and SPL transcription factors

Although transcription factors are traditionally characterized by their DNA‐binding activities, many also function through interactions with other regulatory proteins and multi‐protein complexes. Identifying transcription factor interactors is therefore essential for understanding their molecular mechanisms (Åstrand et al., 2021; Dickey et al., 2026; Gray & Grotewold, 2025; Masiero et al., 2011). Through Y2H screening and targeted BiFC validation, we identified a broad spectrum of SPL8‐associated transcription factors. Among the 26 validated SPL8‐interacting partners, 19 belong to the YABBY, TCP, or SPL families, including four YABBY members, ten TCP members, and five SPL members (Table S1). The enrichment of these three plant‐specific transcription factor families is notable given their established roles in regulating leaf, floral, and anther development (Chen et al., 2014; Nicolas & Cubas, 2016; Sieber et al., 2004; Tao et al., 2024; Wei et al., 2015; Xing et al., 2011). These findings position SPL8 at the center of a transcriptional regulatory hub integrating multiple conserved developmental regulators and suggest that previously unrecognized physical interactions among these families contribute to the coordinated control of anther development.

Several features of the SPL8 interaction network are noteworthy. First, the SPL8‐SPL8 interaction observed in BiFC assays raises the possibility that SPL8 may function as a homodimer, a configuration that often enhances DNA‐binding affinity and target recognition in transcription factors (Boer et al., 2014; Jeong et al., 2009; Li, Chen, et al., 2025; Zhang et al., 2023). Future biochemical studies using purified proteins would help to further define the functional relevance of this self‐interaction. Second, the identification of YAB3 and TCP19 as SPL8 interactors, together with the enrichment of their respective families, suggests that SPL8 may function as a physical bridge between YABBY and TCP transcription factors, facilitating their coordinated assembly into shared transcriptional complexes. Although neither YAB3 nor TCP19 contains a canonical EAR motif (LXLXL/DLNXXP), both interacted with TPL in BiFC assays (Kagale et al., 2010) (Figure S1; Table S2), suggesting that they may participate in a transcriptional repression complex (Plant et al., 2021). A similar mechanism has been reported for SPL/NZZ (Chen et al., 2014; Wei et al., 2015). Third, the absence of detectable interactions between SPL8 and several anticipated candidates, including SPL/NZZ, ROXY1, PI, and AG (Table S2), is equally informative. Although these proteins are well‐established regulators of anther development (Verma, 2019; Xing et al., 2011; Yang et al., 1999), the results suggest that SPL8 functions independently of direct physical association with them, refining current models of the anther transcriptional network (Chang & Ma, 2026; Verma, 2019; Xing et al., 2011).

### *SPL8* regulates the transcriptional expression of *YAB3* and *TCP19*

Inducible expression assays, combined with CHX treatment, demonstrated that *SPL8* directly activates transcription of *YAB3* and *TCP19*, placing these genes downstream of *SPL8* in the regulatory hierarchy. ChIP‐seq analysis identified multiple SPL8‐binding sites within both loci, highlighting these genomic regions as candidates for further examination. Notably, we observed partial discrepancies between the ChIP‐seq and ChIP‐qPCR results: one genomic fragment originally selected as a negative control, which lacked a prominent ChIP‐seq peak, nevertheless exhibited significant SPL8 enrichment in ChIP‐qPCR assays (Figure 4A–D). These differences likely reflect the distinct resolution and analytical characteristics of the two methods. Specifically, ChIP‐seq peak‐calling algorithms apply genome‐wide statistical thresholds that may exclude weaker but biologically relevant binding events, whereas ChIP‐qPCR quantifies enrichment at predefined loci with greater sensitivity (Landt et al., 2012; Park, 2009). Importantly, the validated binding regions were further supported by Y1H and dual‐luciferase assays, confirming their functional relevance. Together, these complementary lines of evidence suggest that genome‐wide ChIP‐seq profiling alone may not fully capture the complete spectrum of SPL8 occupancy and underscore the value of targeted validation approaches.

Interestingly, SPL8 binding within the *TCP19* coding sequence (CDS) represents a non‐canonical mode of transcriptional regulation. Although transcription factors most commonly bind promoter‐proximal regions, intragenic binding has been increasingly recognized as a functionally important regulatory mechanism (Pal & Biswas, 2023). For example, SPL3 binds the *LFY* coding region to regulate its expression, while BZR1 frequently associates with gene bodies to modulate transcriptional elongation (Sun et al., 2010; Yamaguchi et al., 2009; Zhu et al., 2024). The association of SPL8 with the *TCP19* CDS is therefore consistent with this emerging paradigm, suggesting that intragenic binding may contribute to transcriptional regulation beyond promoter occupancy. However, whether SPL8 binding to the *TCP19* CDS influences transcriptional elongation, mRNA processing or stability, or other regulatory processes remains to be determined.

Together, these findings reveal a regulatory circuit in which SPL8 not only physically interacts with YAB3 and TCP19 but also directly controls their transcription. Such dual‐level regulation may facilitate coordinated accumulation of these transcription factors during early anther development and promote the formation of functional regulatory complexes. This multilayered regulatory architecture provides a mechanistic basis for the coordinated activities of *SPL8*, *YAB3*, and *TCP19* in anther development.

### The *SPL8* ‐ *YAB3* / *TCP19* module orchestrates anther development

Multiple lines of genetic evidence support a model in which *SPL8* coordinates anther development through two downstream effectors, *YAB3* and *TCP19*, that perform overlapping yet distinct functions throughout anther development.

*YAB3* functions as a central regulator of anther morphogenesis. Previous studies have shown that YABBY family members cooperate to establish normal anther morphology, as exemplified by the filamentous and clavate stamens of the *fil yab3* double mutant (Siegfried et al., 1999). Although severe vegetative defects in higher‐order *YABBY* mutants display severe vegetative defects that obscure anther phenotypes (Sarojam et al., 2010; Stahle et al., 2009), the consistent clavate anther defects observed in *YAB1/FIL* artificial microRNA lines and in *pSPL8:YAB3‐SRDX* and *pYAB3:YAB3‐SRDX* repressor lines further demonstrate a specific role of *YAB3* in anther morphogenesis (Goldshmidt et al., 2008) (Figure 6H,I; Figure S7e1–f3). Given its prominent abaxial expression in anther primordia (Siegfried et al., 1999) (Figure 5K–N), *YAB3* likely contributes to early anther patterning by regulating abaxial cell fate, analogous to its established role in leaf development (Palermo & Dornelas, 2021; Sarojam et al., 2010).

Stage‐specific repression of YAB3 further revealed that its functions extend beyond early morphogenesis. SRDX‐mediated repression driven by anther‐specific promoters (*DRL1*, *A9*, and *REM22*) demonstrated that *YAB3* is required for tapetum differentiation and microspore development, with the consequent failure of pollen formation. Among these lines, *pREM22:YAB3‐SRDX* exhibited the most severe phenotype, producing anthers with only two pollen sacs instead of the typical four (Figure S9c,j). This observation suggests that *YAB3* may participate not only in anther patterning but also in determining the number of pollen sacs, raising the intriguing possibility that it contributes to an evolutionarily conserved mechanism controlling anther architecture (Åstrand et al., 2021; Romanova et al., 2021). Conversely, excessive *YAB3* activity also profoundly altered floral development. Constitutive overexpression of *YAB3* in *pUBQ10:YAB3‐3HA* plants resulted in supernumerary stamens at the expense of the carpel/gynoecium, closely resembling the phenotype of the *superman (sup)* mutant (Bowman et al., 1992). According to the ABC model of floral organ identity, this carpel‐to‐stamen homeotic transformation implies ectopic activation of B‐class genes such as *APETALA3* (*AP3*) and *PISTILLATA* (*PI*) (Bowman & Moyroud, 2024; Irish, 2017). Intriguingly, SUP represses B‐function gene expression, likely by recruiting the TOPLESS (TPL) corepressor via its EAR motif (Prunet et al., 2017; Wokasch et al., 2025). Nevertheless, whether *YAB3* regulates *AP3* and *PI* through the same pathway, acts in parallel with *SUP*, or functions independently to modulate B‐class gene expression remains to be determined. Together, these findings establish *YAB3* as a multifunctional regulator of stamen and anther development, coordinating floral organ identity, pollen sac formation, early anther patterning, tapetum differentiation, and microspore development.

*TCP19* constitutes a second functional branch of this regulatory module. Unlike most other members of the TCP family, *TCP19* remains largely functionally uncharacterized (Viola et al., 2023; Wang et al., 2025). Although CRISPR/Cas9‐generated *tcp19* mutants exhibit no obvious developmental defects, likely due to functional redundancy with closely related paralogs such as *TCP9* and *TCP20*, SRDX‐mediated repression uncovered clear roles for *TCP19* during anther development.

Unlike the *pDRL1:YAB3‐SRDX* and *pDRL1:SPL8‐SRDX* lines, *pDRL1:TCP19‐SRDX* plants exhibited meiotic arrest and mild tapetal enlargement (Figure 7N; Figure S8d3,d4), a phenotype partially reminiscent of that observed in *dyt1*, *tdf1*, *ams*, *myb33 myb65*, and *roxy1 roxy2* mutants (Millar & Gubler, 2005; Sorensen et al., 2003; Xing & Zachgo, 2008; Zhang et al., 2006; Zhu et al., 2008). The meiotic defect suggests that *TCP19* is required for the progression of meiosis in microspore mother cells, whereas the tapetal enlargement points to an additional role in restricting tapetal growth. This phenotype was consistently recapitulated in *pREM22:TCP19‐SRDX* lines (Figure S9m–q), further supporting a role for *TCP19* during early anther development. The phenotypic resemblance to these known anther regulators suggests that *TCP19* may act in a common genetic pathway or at a similar regulatory node during early anther development, though the precise hierarchical relationship remains to be determined.

Beyond its roles in tapetum and meiosis regulation, *TCP19* also appears to function in stamen organ identity. This is supported by the presence of carpelloid anthers in *TCP19‐SRDX* lines (Figures S7h2–i3 and S9r–t), a phenotype reminiscent of the floral homeotic mutant *ap3* (Bowman et al., 1989). As proposed for *YAB3*, *TCP19* may function through interactions with the *AP3/PI* regulatory pathway, suggesting that *TCP19* and *YAB3* contribute cooperatively, potentially through shared downstream targets, to regulate floral organ identity.

*TCP19* may also contribute to early anther patterning. The occasional reduction in pollen sac number observed in *pREM22:TCP19‐SRDX* lines resembles phenotypes observed in *spl8‐1* and *pREM22:YAB3‐SRDX* plants (Unte et al., 2003) (Figure S9g–k,u). These shared developmental defects suggest that *TCP19*, together with *YAB3* and *SPL8*, coordinately regulates early anther morphogenesis, in addition to its roles in maintaining anther identity and promoting meiosis and tapetum function.

*SPL8* functions in anther development (Unte et al., 2003; Xing et al., 2010), as further confirmed by the *pDRL1:SPL8‐SRDX* phenotypes (Figure 7E–H). Interestingly, *spl8* mutants exhibit defects in meiotic progression, whereas the SRDX lines undergo relatively normal meiosis but display impaired microspore release from tetrads. These findings suggest that *SPL8* has a distinct role in promoting microspore release. The absence of meiotic defects in the *pDRL1:SPL8‐SRDX* lines may reflect differences in the spatiotemporal expression domains of the *DRL1* promoter relative to endogenous *SPL8*, or it may indicate that the meiotic function of *SPL8* is mediated through mechanisms that are not effectively targeted by the SRDX‐mediated repression.

Genetic analyses further showed that the *spl8‐1 yab3 tcp19* triple mutant phenocopied *spl8‐1* mutant, suggesting that *YAB3* and *TCP19* function downstream of *SPL8*. This epistatic relationship is consistent with previous observations of phenotypic convergence between *SPL*‐deficient and *TCP*‐deficient mutants. In *spl8* mutants, reduced activity of miR156‐targeted *SPL* members (*SPL2*/*9*/*10*/*11*/*13*/*15*), as well as disruption of miR319‐targeted *TCP* genes, results in severe male sterility associated with the absence of pollen sacs, suggesting a potential genetic connection between the *SPL* and *TCP* regulatory pathways (Tao et al., 2024; Xing et al., 2010). Together, these findings provide functional evidence for a model in which *SPL8* acts upstream of *YAB3* and *TCP19* to coordinate an integrated regulatory module controlling anther development.

### Mechanistic insights into the *SPL8* ‐ *YAB3* / *TCP19* module in anther development

Based on the biochemical, genetic, and phenotypic evidence presented in this study, we propose a working model for the *SPL8*–*YAB3*/*TCP19* module that integrates transcriptional regulation and protein–protein interactions to control anther development (Figure 8).

**Figure 8:**
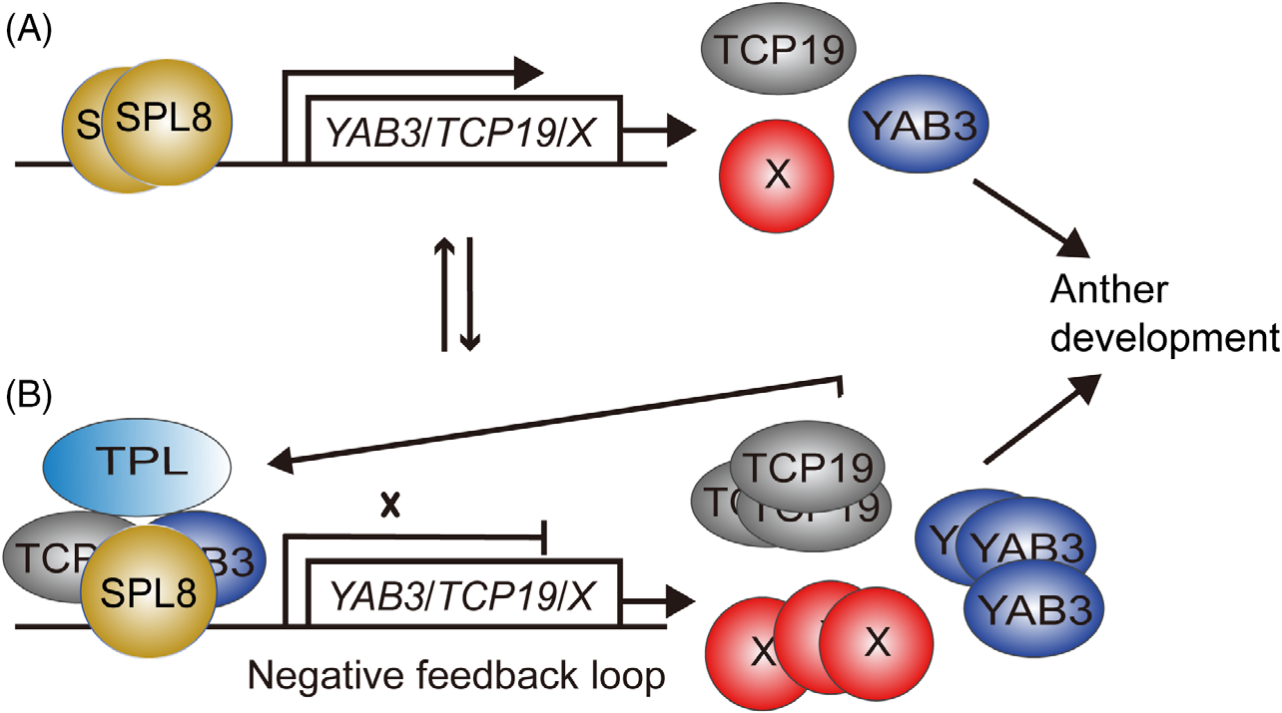
Proposed model for *SPL8*‐mediated regulation of *YAB3* and *TCP19* during anther development. This working model illustrates the *SPL8*‐centered regulatory network. In the active state (A), SPL8 homodimers bind GTAC‐containing cis‐elements in the regulatory regions of *YAB3*, *TCP19*, and other potential downstream genes (X), thereby activating their transcription. The resulting accumulation of YAB3, TCP19, and other target proteins promotes normal anther development. As YAB3 and TCP19 accumulate, they initiate a negative feedback loop (B). Individually or cooperatively, these proteins interact with SPL8 and recruit TPL co‐repressors to form transcriptional repressor complexes that suppress the expression of *YAB3* and *TCP19*, thereby reducing protein accumulation. Upon dissociation of the repressor complexes, SPL8 is released to re‐form homodimers and reactivate target gene expression, restoring the active state (A). This reversible negative feedback mechanism fine‐tunes SPL8 activity and maintains the homeostasis of anther developmental gene expression, thereby ensuring proper anther development in *A. thaliana*.

In this model, the module transitions between two functional states. In State A, SPL8, likely functioning as a homodimer, binds to cis‐regulatory elements of *YAB3*, *TCP19*, and other downstream anther‐related genes to activate their transcription. As YAB3 and TCP19 proteins accumulate in the nucleus, they interact individually with SPL8, forming SPL8–YAB3 and SPL8–TCP19 heterodimers, and may also associate with each other to promote the assembly of higher‐order regulatory complexes. These complexes subsequently recruit the transcriptional corepressor TPL, resulting in the formation of repressive complexes.

In State B, these repressive complexes attenuate the expression of *YAB3*, *TCP19*, and other *SPL8* target genes. Because both *YAB3* and *TCP19* are transcriptional targets of *SPL8*, their protein products may participate in feedback regulation by limiting *SPL8*‐dependent transcription through the formation of SPL8‐containing repressor complexes. This negative‐feedback mechanism provides a means to constrain the duration and magnitude of *SPL8*‐mediated transcriptional activation.

The dynamic equilibrium between these two states is likely determined by the abundance of YAB3 and TCP19 proteins. When their nuclear levels decrease due to reduced transcription and protein turnover, repressive complexes may dissociate, allowing SPL8 to reform active homodimers and reinitiate the transcription of *YAB3*, *TCP19*, and other downstream targets, thereby restoring State A. Such a self‐regulatory circuit may ensure that the expression of these key regulators is maintained within an optimal range, thereby preventing insufficient or excessive transcriptional activity.

Several aspects of this model require further experimental validation. First, the proposed dual‐layer feedback, in which YAB3 and TCP19 independently contribute to repression and may further influence complex assembly through their mutual interaction, could provide robustness and flexibility to the regulatory network. This feature may be particularly important for coordinating the precise temporal and spatial transcriptional changes required during anther development (Gray & Grotewold, 2025). Second, although interaction assays demonstrate that YAB3 and TCP19 can physically associate with SPL8 and with each other, the stoichiometry, composition, and assembly dynamics of the proposed multi‐protein complexes remain unclear. Whether these complexes assemble cooperatively or competitively, and whether their composition changes across developmental stages or cell types, are important questions for further investigation. Third, recruitment of TPL as a corepressor is supported by BiFC evidence showing interactions between YAB3–TPL and TCP19–TPL (Figure S1). Direct biochemical validation of TPL recruitment in native anther tissues, for example through co‐immunoprecipitation using anther extracts or in vitro pull‐down assays using purified proteins, would further strengthen this model.

In summary, the *SPL8*–*YAB3*/*TCP19* module may function through a self‐balancing negative‐feedback mechanism that fine‐tunes the spatiotemporal expression of *YAB3*, *TCP19*, and other downstream anther‐associated genes, thereby ensuring proper anther development in Arabidopsis. This regulatory framework provides a mechanistic explanation for the coordinated functions of these transcription factors and illustrates how transcriptional regulation and protein–protein interactions can be integrated to achieve developmental precision.

## EXPERIMENTAL PROCEDURES

### Plant materials and growth conditions

*Nicotiana benthamiana* seeds were sown in a 1:1 (v/v) mixture of unfertilized nutrient soil and vermiculite. Seedlings were transplanted 2 weeks after sowing and grown for an additional 3–4 weeks before use in experiments.

*Arabidopsis thaliana* ecotype Columbia‐0 (Col‐0) was used as the wild type. Laboratory‐maintained plant materials included the *spl8‐1* mutant and the transgenic line *p35S:SPL8‐GR*. The *yab3* mutant (SAIL_256_D07/N812017) was obtained from the Nottingham Arabidopsis Stock Centre (NASC). Seeds were surface sterilized, stratified on moist filter paper at 4°C in the dark for 2 days, and then sown in the same soil substrate.

All plants were grown in a greenhouse at 22–24°C and 60–70% relative humidity under a 16 h light/8 h dark photoperiod with a light intensity of 120 μmol m^−2^ sec^−1^.

### Yeast two‐hybrid (Y2H) assay

Y2H assays were conducted using the GAL4‐based Matchmaker system. To exclude bait autoactivation and toxicity, Y2HGold yeast cells were co‐transformed with the bait plasmids pGBKT7‐SPL8 or pGBKT7‐YAB3 together with the empty prey vector pGADT7. The bait constructs were subsequently co‐transformed with the corresponding prey plasmids (pGADT7‐YAB3 or pGADT7‐TCP19). The combinations pGBKT7‐53/pGADT7‐T and pGBKT7‐Lam/pGADT7‐T were used as positive and negative controls, respectively.

Transformants were first selected on SD/‐Leu/‐Trp (DDO) medium and subsequently screened on SD/‐Leu/‐Trp/‐His (TDO) and SD/‐Leu/‐Trp/‐His/‐Ade (QDO) media. Positive protein–protein interactions were confirmed by serial dilution spot assays. Screening of the Arabidopsis transcription factor library was performed by Shanghai OE Biotech Co., Ltd, Shanghai, China.

### Bimolecular fluorescence complementation (BiFC) assay

Recombinant BiFC constructs were introduced into *Agrobacterium tumefaciens* strain GV3101 using the freeze–thaw method. Positive colonies were cultured overnight in LB medium at 28°C, pelleted by centrifugation, and resuspended in infiltration buffer (10 mm MES pH 5.7, 10 mm MgCl_2_, and 150 μm acetosyringone) to an OD_600_ of 0.8. The suspensions were incubated in the dark at room temperature for 2 h before infiltration.

Equal volumes of *Agrobacterium* suspensions harboring complementary BiFC constructs were mixed and infiltrated into fully expanded leaves of 5‐week‐old *N. benthamiana* using needleless syringes. Following infiltration, plants were incubated in darkness for 12 h and then returned to normal growth conditions for 2–4 days. YFP fluorescence signals were observed using either an epifluorescence microscope or a Zeiss LSM710 confocal laser scanning microscope (Carl Zeiss Microscopy GmbH, Jena, Germany).

### Fluorescence resonance energy transfer–fluorescence lifetime imaging microscopy (FRET–FLIM)

The pFRET‐2in1‐CC constructs (pFRET‐2in1‐CC‐TCP19‐mCherry‐SPL8‐GFP, pFRET‐2in1‐CC‐YAB3‐mCherry‐SPL8‐GFP, and pFRET‐2in1‐CC‐YAB3‐mCherry‐TCP19‐GFP) were transiently expressed in *N. benthamiana* leaves via *Agrobacterium*‐mediated transformation as described for the BiFC assay. In each construct, GFP served as the donor fluorophore and mCherry as the acceptor fluorophore.

FLIM imaging was performed using a Leica SP8 confocal microscope equipped with a time‐correlated single‐photon counting (TCSPC) system. The GFP donor was excited using a 488‐nm pulsed laser, and fluorescence emission was collected between 490 and 530 nm. mCherry fluorescence was detected between 565 and 610 nm following excitation at 561 nm to confirm acceptor expression. Imaging parameters were optimized to minimize spectral bleed‐through between donor and acceptor channels.

Fluorescence lifetime data were acquired using SymPhoTime 64 v2.1 (PicoQuant, Berlin, Germany). Images were collected until sufficient photon counts were obtained for reliable lifetime fitting. Donor fluorescence lifetimes were calculated from fluorescence decay curves, and pseudocolor lifetime maps were generated to visualize spatial variations in fluorescence lifetime. A reduction in the fluorescence lifetime of GFP in the presence of the mCherry‐tagged acceptor, relative to the control (bait‐GFP + free mCherry), was interpreted as evidence of FRET, indicating a direct protein–protein interaction.

### Chemical and heat stress treatments

For glucocorticoid induction, dexamethasone (DEX) and cycloheximide (CHX) stock solutions (10 mm) were prepared in absolute ethanol and stored at −20°C. Working solutions (10 μm) were freshly prepared in distilled water, with 0.1% (v/v) ethanol as the mock control. Two‐week‐old wild‐type (Col‐0) and *35S:SPL8‐GR* transgenic seedlings were sprayed with mock, DEX, CHX, or DEX + CHX solutions. After 6 h under normal growth conditions, leaf tissues were harvested, immediately frozen in liquid nitrogen, and stored at −80°C until further analysis.

For heat stress treatment, 2‐week‐old *pHSP18.2:GUS* transgenic seedlings grown at 22–24°C were transferred to 37°C for 2 h and then returned to 22–24°C for recovery. Samples were collected at 2, 4, 6, 8, 10, 12, and 24 h after recovery for histochemical GUS staining. Based on the staining results, the 6‐h recovery time point was selected for subsequent experiments. Two‐week‐old *pHSP18.2:GFP* and *pHSP18.2:SPL8‐GFP* transgenic seedlings were subjected to the same heat treatment (37°C for 2 h followed by 6 h recovery at 22–24°C), harvested, frozen in liquid nitrogen, and stored at −80°C for RT‐qPCR and chromatin immunoprecipitation (ChIP) analyses.

### Chromatin immunoprecipitation sequencing (ChIP‐seq) and ChIP‐qPCR

Chromatin was isolated from heat‐treated *pHSP18.2:SPL8‐GFP* transgenic seedlings, cross‐linked, and immunoprecipitated using GFP‐Trap® beads according to the manufacturer's instructions. The purified DNA was subjected to high‐throughput sequencing. Heat‐treated *pHSP18.2:GFP* transgenic seedlings were processed in parallel and used as a negative control to eliminate non‐specific background signals.

Sequencing reads were mapped to the Arabidopsis reference genome, and peak calling was performed separately for the SPL8‐GFP and GFP control samples using MACS2 (v2.2.1) with the corresponding input libraries as references (*q* < 0.05). Since downstream analysis focused specifically on the *YAB3* and *TCP19* loci, we manually inspected these regions using IGV (v2.17.4) to select candidate fragments for ChIP‐qPCR primer design (Table S4).

For ChIP‐qPCR validation, three independent biological replicates were analyzed. Primers were designed to amplify promoter regions and coding regions containing predicted SPL8‐binding sites (Table S5). ChIP DNA was used as the template for quantitative PCR. Enrichment of immunoprecipitated DNA was determined relative to the IgG control using the 2^−ΔΔ*Ct*^ method, with the IgG‐normalized values set to 1.0. Significant enrichment relative to the IgG control was considered evidence of specific SPL8 binding to the corresponding genomic regions.

### Yeast one‐hybrid (Y1H) assay

For bait construction, the linearized pAbAi‐YAB3 vector containing a 1208‐bp distal promoter fragment of *YAB3* and the pAbAi‐TCP19 vector containing a 747‐bp coding‐region fragment of *TCP19* were individually transformed into Y1HGold yeast cells and selected on SD/‐Ura medium. Single colonies were used to prepare competent yeast cells and were transformed with the empty pGADT7 vector to evaluate bait toxicity and autoactivation. Transformants were grown on SD/‐Leu medium supplemented with increasing concentrations of aureobasidin A (AbA). Normal growth on SD/‐Leu medium confirmed successful transformation and the absence of bait toxicity, whereas autoactivation assays established 1000 ng/ml AbA as the optimal selection concentration for both bait strains.

To examine SPL8 binding, pGADT7‐SPL8 was individually co‐transformed into each bait strain. Serially diluted yeast cultures (10^−1^–10^−4^) were spotted onto SD/‐Leu plates with or without 1000 ng/ml AbA. Growth on AbA‐containing medium indicated binding of SPL8 to the corresponding *YAB3* promoter fragment or *TCP19* genomic fragment. The pGADT7‐rec53/pAbAi‐p53 and the empty pGADT7/pAbAi‐p53 combinations served as positive and negative controls, respectively. All Y1H assays were performed in collaboration with Wuhan GeneCreate Biological Engineering Co., Ltd, Wuhan, China.

### Dual‐luciferase reporter assay

Reporter and effector constructs were co‐infiltrated into *N. benthamiana* leaves via *A. tumefaciens* strain GV3101. After incubation under normal growth conditions for 48–72 h, infiltrated leaf tissues were harvested for measurement of firefly luciferase (LUC) and *Renilla* luciferase (REN) activities using the Dual‐Luciferase® Reporter Assay System (Promega, Madison, WI, USA). Relative promoter activity was calculated as the ratio of LUC to REN activity, with the empty effector and reporter vectors serving as the negative control. All dual‐luciferase assays were performed in collaboration with Wuhan GeneCreate Biological Engineering Co., Ltd.

### Plasmid construction and generation of transgenic plants

All plasmids were constructed using conventional restriction enzyme‐mediated cloning or Gateway BP/LR recombination (File S1). Target CDS, promoter fragments, and other regulatory sequences were amplified from *A. thaliana* Col‐0 cDNA or genomic DNA. Synthetic DNA fragments were synthesized by Sangon Biotech (Shanghai, China). All constructs were validated by Sanger sequencing before use.

For protein–protein and protein–DNA interaction assays, the full‐length CDSs of *SPL8*, *YAB3*, and *TCP19* were individually subcloned into the pGBKT7 and pGADT7 vectors for Y2H assays or into the pAbAi vector for Y1H assays. The Y1H bait constructs contained either a 1208‐bp distal promoter fragment of *YAB3* or a 747‐bp partial coding‐region fragment of *TCP19*. For BiFC and FRET–FLIM analyses, the corresponding coding sequences were cloned into the pEarleyGate and pFRETgc‐2in1 vectors, respectively. For dual‐luciferase reporter assays, the same *YAB3* promoter fragment and *TCP19* coding‐region fragment were inserted into the reporter vector pGreenII 0800‐LUC, whereas the full‐length *SPL8* coding sequence was cloned into the effector vector pGreenII 62‐SK.

Promoter‐GUS reporter constructs and promoter‐protein GFP translational fusion constructs were generated by cloning the corresponding genomic fragments into binary vectors. CRISPR/Cas9 constructs targeting *TCP19* were generated by inserting guide RNA sequences into the HBp402 vector, followed by Gateway LR recombination with pDe‐Cas9. SRDX repressor constructs were generated by fusing the target gene's CDS to the SRDX repressive motif under the control of endogenous, ubiquitous, or tissue‐specific promoters.

All recombinant plasmids were introduced into *A. tumefaciens* strain GV3101 for either transient expression in *N. benthamiana* leaves or stable transformation of Arabidopsis Col‐0 or *spl8‐1* mutants using the floral dip method. Transgenic plants were screened based on Basta resistance or Fast Red fluorescence (Shimada et al., 2010), and positive lines were further confirmed by PCR.

### RNA extraction and RT‐qPCR analysis

Total RNA was extracted from Arabidopsis tissues using RNAiso Plus reagent (Takara, Japan) and treated with DNase I to remove residual genomic DNA. RNA quality and concentration were assessed by agarose gel electrophoresis and NanoDrop 2000 spectrophotometry (Thermo Fisher Scientific, Waltham, MA, USA).

First‐strand cDNA was synthesized from 1 μg of total RNA using the HiScript® III RT SuperMix for qPCR (+gDNA wiper) Kit (Vazyme, Nanjing, China) according to the manufacturer's instructions. A no‐reverse transcriptase control was included to verify the absence of genomic DNA contamination.

RT‐qPCR was performed using a LightCycler 480 II Real‐Time PCR System (Roche Diagnostics GmbH, Mannheim, Germany) with SYBR qPCR Master Mix (Promega, Beijing, China) and gene‐specific primers listed in Table S5. Melting curve analysis was conducted to confirm amplification specificity. Relative transcript levels were calculated using the 2^−ΔΔ*Ct*^ method with *ACT2* as the internal reference. Each experiment included at least three independent biological replicates and three technical replicates.

### RNA *in situ* hybridization

RNA *in situ* hybridization was performed as previously described (Xing et al., 2005). A 687‐bp fragment covering the *SPL8* 5′ UTR–first exon junction, as well as the full‐length CDSs of *YAB3* and *TCP19*, was amplified using gene‐specific primers appended with T3/T7 promoter sequences (Table S5). Antisense RNA probes were synthesized using T7 RNA polymerase (Roche Diagnostics GmbH, Mannheim, Germany) according to the manufacturer's instructions and hydrolyzed before use. Hybridization was performed overnight at 50 °C in a drying oven (GZX‐9246MBE, BOXUN, Shanghai, China).

### Histology and microscopy

Inflorescences or open flowers from 4‐ to 5‐week‐old Arabidopsis plants were fixed in 4% (v/v) glutaraldehyde at 4°C for at least 24 h. Samples were subsequently dehydrated, embedded in paraffin, and sectioned transversely at a thickness of 5 μm using a Leica RM2235 microtome (Leica Biosystems Nussloch GmbH, Nussloch, Germany). Sections were deparaffinized, rehydrated, and stained with 0.05% (w/v) toluidine blue O for 1–3 min at room temperature. After three washes with distilled water, sections were air‐dried and mounted with neutral balsam (Solarbio, Beijing, China) under a fume hood. Images were captured using a Leica DFC450 bright‐field microscope (Leica Microsystems, Mannheim, Germany).

### Data analysis

Statistical analyses were performed using GraphPad Prism (version 9.0; GraphPad Software, San Diego, CA, USA). Gene expression data were analyzed by one‐way analysis of variance (anova) followed by Tukey's honestly significant difference (HSD) post hoc test for multiple comparisons. Other datasets were analyzed using two‐tailed Student's *t*‐tests, with Welch's correction applied when variances were unequal.

Data are presented as the mean ± standard error (SE) from at least three independent biological replicates. Asterisks indicate statistically significant differences relative to the corresponding control groups: **P ≤ 0.01; ***P ≤ 0.001; ****P ≤ 0.0001. ns, not significant (*P* > 0.05).

## GENE LOCUS IDS

*A9* (AT5G07230), *ACT2* (AT3G18780), *DRL1* (ATG35420), *DYT1* (AT4G21330), *HSP18.2* (AT5G59720), *REM22* (AT3G17010), *SERK2* (AT1G34210), *TDF1* (AT3G28470).

## AUTHOR CONTRIBUTIONS

SX conceptualized and designed the study. TL conducted the experiments, collected the data, and performed the formal analysis. LZ assisted with the FRET‐FILM experiments and contributed to the interpretation of the data. TL drafted the original manuscript. SX provided critical review and editing of the manuscript. All authors reviewed the final version and approved its submission.

## CONFLICT OF INTEREST

All authors declare that they have no conflicts of interest regarding the publication of this manuscript.

## Supporting information


**File S1.** Plasmid construction methods.
**Figure S1.** Protein–protein interactions validated via BiFC assays.
**Figure S2.** *DRL1*, *A9*, and heat‐inducible *HSP18.2* promoter activities in GUS and GFP reporter lines.
**Figure S3.** Gene structures and fragments used for yeast one‐hybrid (Y1H) assays.
**Figure S4.** Schematic diagram of effector and reporter constructs for dual‐luciferase assays.
**Figure S5.** GUS reporter and GFP fusion analysis of *SPL8*, *YAB3*, and *TCP19* expression patterns in inflorescences.
**Figure S6.** Expression analysis of *SPL8*, *YAB3*, and *TCP19* in different mutant backgrounds.
**Figure S7.** Floral phenotypes of various genotypes.
**Figure S8.** Anther histology of transgenic lines expressing SRDX fusions driven by the *DRL1* promoter.
**Figure S9.** Histological analysis of anther cross‐sections of transgenic lines expressing *YAB3‐SRDX* or *TCP19‐SRDX* under the *REM22* promoter.

**Table S1.** Putative SPL8‐interacting transcription factors.
**Table S2.** List of BiFC‐tested interactions in this study.
**Table S3.** ChIP‐seq peak counts for SPL8 and GFP control.
**Table S4.** Primer design information for ChIP‐qPCR.
**Table S5.** The primers used in this study.

## Data Availability

The data supporting the findings of this study are presented in the main text and the supplementary material of this article. Additional raw data are available from the corresponding author upon reasonable request.
