# Supplementary material for: The SPL8‐YAB3/TCP19 module integrates transcriptional and protein–protein regulatory mechanisms to orchestrate anther development in Arabidopsis
Source: Plant J. 2026 Oct 11;128(1):e71160. doi: 10.1111/tpj.71160 (PMC13655359; doi:10.1111/tpj.71160)
Supplement: Supplementary file 1 [file TPJ-128-0-s002.pdf]

Supplemental file S1

Plasmid construction methods

All plasmids were constructed using standard molecular cloning techniques, including restriction enzyme-mediated ligation and Gateway recombination (BP/LR reactions). Gene fragments were PCR-amplified from either wild-type *Arabidopsis thaliana* (Col-0) inflorescence cDNA or genomic DNA. Synthetic DNA fragments were obtained from Sangon Biotech (Shanghai, China). The integrity of all final constructs was confirmed via restriction enzyme digestion and Sanger sequencing.

Yeast two-hybrid vector construction

For yeast two-hybrid (Y2H) assays, the full-length coding sequences (CDSs) of *SPL8*, *YAB3*, and *TCP19* were PCR-amplified from *Arabidopsis* wild-type Col-0 inflorescence cDNA using gene-specific primers that incorporated restriction sites (Table S5). The 1,002-bp *SPL8* and 723-bp *YAB3* CDSs were cloned into the bait vector pGBKT7 via *Nde*I and *Sma*I sites, whereas the 723-bp *YAB3* and 882-bp *TCP19* CDSs were inserted into the prey vector pGADT7 using *Nde*I and *Xho*I sites. Following amplification, PCR products were resolved on 1% (w/v) agarose gels, and bands of the expected sizes were excised and gel-purified. The purified inserts and corresponding vectors were subjected to double digestion with the appropriate restriction enzymes, re-purified, and ligated overnight at 4°C. The ligation mixtures were then transformed into *E. coli* DH5α competent cells, which were subsequently plated on LB agar supplemented with the appropriate antibiotics and incubated overnight at 37°C. Positive clones were identified via colony PCR. The resulting constructs were designated pGBKT7-SPL8, pGBKT7-YAB3, pGADT7-YAB3, and pGADT7-TCP19.

Yeast one-hybrid (Y1H) vector construction

For the yeast one-hybrid (Y1H) assays, the full-length coding sequence (CDS) of *SPL8* (1,002-bp) was amplified from inflorescence cDNA using gene-specific primers (Table S5) and cloned into the pGADT7 vector via *Nde*I and *Xho*I restriction sites, generating the prey/activator construct pGADT7-SPL8. For the reporter constructs, a 747-bp partial coding region of *TCP19* and a 1,208-bp distal promoter fragment of *YAB3*—both identified as containing putative SPL8-binding sites based on ChIP analysis—were obtained through gene synthesis. These fragments were then individually inserted into the pAbAi vector using *Kpn*I and *Xho*I sites, resulting in the reporter constructs pAbAi-TCP19 and pAbAi-YAB3, respectively.

Bimolecular fluorescence complementation (BiFC) vector construction

For BiFC assays, the full-length CDSs of candidate interactors were amplified from Col-0 inflorescence cDNA using gene-specific primers appended with attB1 and attB2 sites (Table S5). The resulting PCR products were first cloned into the entry vector pDONR207 via BP recombination (BP Clonase II, Invitrogen) and subsequently transferred into Gateway-compatible BiFC destination vectors—pEarleyGate201-C-nYFP, pEarleyGate202-C-cYFP, and pEarleyGate203-nYFP-N—through LR recombination (LR Clonase II, Invitrogen), yielding C-terminal or N-terminal fusions with the N-terminal half

(nYFP) or C-terminal half (cYFP) of YFP. Previous studies and our unpublished data have demonstrated that the SPL8-GFP fusion protein (C-terminal GFP tag), but not GFP-SPL8 (N-terminal GFP tag), fully rescues the defective phenotype of the *spl8-1* mutant (Xing et al., 2010). Accordingly, for BiFC detection, the nYFP or cYFP fragment was fused to the C-terminus of SPL8, whereas both N- and C-terminal fusions with each YFP half were generated for all other candidate interactors.

Fluorescence resonance energy transfer-fluorescence lifetime imaging microscopy (FRET-FLIM) vector construction

For FRET-FLIM assays, full-length CDSs of *SPL8*, *YAB3*, and *TCP19* (999-bp, 720-bp, and 879-bp, respectively) were amplified from Col-0 inflorescence cDNA using gene-specific primers carrying attB recombination sites (attB1/attB4 or attB2/attB3), with stop codons omitted to ensure in-frame fusions with fluorescent proteins. The resulting PCR products were sequentially recombined into the Gateway entry vectors pDONR221-P1P4 and pDONR221-P2P3 via BP reactions (BP Clonase II, Invitrogen), followed by LR recombination (LR Clonase II, Invitrogen) into the FRET destination vector pFRETgc-2in1-CC. This yielded the final expression constructs: pFRETgc-2in1-CC-TCP19-mCherry-SPL8-GFP, pFRETgc-2in1-CC-YAB3-mCherry-SPL8-GFP, and pFRETgc-2in1-CC-YAB3-mCherry-TCP19-GFP.

Dual-luciferase reporter (DLR) and effector vector construction

For DLR assays, a 1,208-bp distal promoter fragment of *YAB3* and a 747-bp partial coding region fragment of *TCP19* were chemically synthesized. The *YAB3* fragment was flanked with *Hind*III and *BamH*I sites, while the *TCP19* fragment was flanked with *Hind*III and *Bgl*II sites. These fragments were then independently cloned upstream of the firefly luciferase (LUC) reporter gene in the pGreenII 0800-LUC vector via the corresponding *Hind*III and *BamH*I restriction sites, yielding the reporter constructs pGreenII 0800-YAB3 and pGreenII 0800-TCP19, respectively. For the effector construct, the full-length CDS of *SPL8* (1,002-bp) was amplified from Col-0 inflorescence cDNA using gene-specific primers appended with *Spe*I and *Bgl*II sites (Table S5). The PCR product was double-digested and ligated into the pGreenII 62-SK vector (*Spe*I/*BamH*I), generating the effector plasmid pGreenII 62-SK-SPL8.

Promoter–GUS reporter constructs

Promoter fragments of *HSP18.2* (931-bp), *SPL8* (2,124-bp), *YAB3* (5,411-bp), *TCP19* (4,015-bp), *DRL1* (2,004-bp), and *A9* (1,461-bp) were amplified from Col-0 genomic DNA using gene-specific primers that incorporated restriction enzyme sites (*HSP18.2*: *Xba*I/*Sma*I; *SPL8*: *Xba*I/*Sma*I; *YAB3*: *Avr*II/*Sma*I; *TCP19*: *Nhe*I/*Sma*I; *DRL1*: *Xba*I/*Sma*I; *A9*: *Spe*I/*Sma*I). Each promoter fragment corresponded to the genomic region immediately upstream of the respective translational start codon (ATG). The purified PCR products were subjected to double digestion with the corresponding restriction enzymes and subsequently cloned into the binary vector pGPTV-BAR, upstream of the β-glucuronidase (*uidA*) reporter gene. This generated the constructs pHSP18.2:GUS, pSPL8:GUS, pYAB3:GUS, pTCP19:GUS, pDRL1:GUS, and pA9:GUS, respectively.

GFP fusion constructs

Genomic fragments encompassing the coding regions and upstream promoter sequences of *SPL8*, *YAB3*, and *TCP19* were amplified from Col-0 genomic DNA. The following genomic intervals were used: a 3,000-bp promoter region and a 1,522-bp coding/intron segment for *SPL8*; a 5,411-bp promoter and a 2,283-bp coding/intron segment for *YAB3*; and a 4,015-bp promoter and an 879-bp intronless coding sequence for *TCP19*. For all three genes, the amplified coding and intron regions spanned from the ATG start codon to the sequence immediately upstream of the stop codon, thereby allowing C-terminal in-frame fusions with GFP.

Gene-specific primers incorporating restriction sites for directional cloning were designed as follows: *Stu*I and *Nhe*I for the *SPL8* genomic fragment; *Sma*I for the *YAB3* promoter and *StuI*/*Nhe*I for its coding/intron fragment; and *Sma*I and *Asc*I for the *TCP19* genomic fragment. All restriction sites were appended to the 5′ ends of the primers. Following PCR amplification, the purified fragments were double-digested with the corresponding enzymes and ligated in-frame into a modified plant binary vector upstream of the GFP coding sequence, generating the translational fusion constructs pSPL8:SPL8-GFP, pYAB3:YAB3-GFP, and pTCP19:TCP19-GFP.

pHSP18.2:GFP and pHSP18.2:SPL8-GFP constructs

To generate pHSP18.2:GFP, a 931-bp *HSP18.2* promoter fragment was amplified from Col-0 genomic DNA using primers incorporating *Stu*I and *Xba*I restriction sites. The purified PCR product was digested with *Stu*I and *Xba*I and ligated into the corresponding sites of the existing vector pAtSPL8:GFP, replacing the *SPL8* promoter to generate pHSP18.2:GFP. To generate pHSP18.2:SPL8-GFP, the full-length *SPL8* CDS (without stop codon) was amplified from Col-0 inflorescence cDNA using primers containing *Xba*I sites. The PCR product was digested with *Xba*I and ligated into *Xba*I-linearized pHSP18.2:GFP, yielding the final construct pHSP18.2:SPL8-GFP.

CRISPR/Cas9 construct for *tcp19* mutants

To generate CRISPR/Cas9-mediated knockout constructs for *TCP19*, two 20-bp protospacer sequences targeting the N- and C-terminal regions of the gene were designed (Table S5). Complementary oligonucleotides for each protospacer were annealed (95°C for 5 min, followed by gradual cooling to room temperature over 20 min) to generate double-stranded inserts. For vector assembly, the first protospacer was ligated into *Bbs*I-digested HBp402, and the resulting intermediate (HBp402-*tcp19*-1) was verified by colony PCR and Sanger sequencing. The second protospacer was then inserted into *BsmB*I-digested HBp402-*tcp19*-1, yielding HBp402-*tcp19*-2, which was again sequence-verified. Finally, the dual-sgRNA cassette was recombined with the destination vector pDe-CAS9-ccdb-incl, generating the final CRISPR/Cas9 construct pDe-Cas9-*tcp19*.

pUBQ10:SPL8/YAB3/TCP19-3HA and pUBQ10:YAB3/TCP19-SRDX constructs

To generate pUBQ10:SPL8-3HA, pUBQ10:YAB3-3HA and pUBQ10:TCP19-3HA, the coding sequences of *SPL8*, *YAB3* and *TCP19* (without stop codons) were amplified from wild-type Col-0 inflorescence cDNA and cloned into pDONR207 via BP reactions (BP Clonase II,

Invitrogen). The resulting entry clones were then recombined into the destination vector pUB-3HA-DEST via LR recombination (LR Clonase II, Invitrogen). For the dominant repression constructs, the YAB3-SRDX and TCP19-SRDX cassettes were chemically synthesized (Sangon Biotech, Shanghai, China) and recombined into pDONR207 via BP reactions to generate the entry clones pDONR207-YAB3-SRDX and pDONR207-TCP19-SRDX. These entry vectors were subsequently transferred into the destination vector pUB-DEST via LR recombination, yielding pUBQ10:YAB3-SRDX and pUBQ10:TCP19-SRDX. To enable fluorescent seed selection, the pOLE:OLE-TagRFP fragment was excised from a pDe-CAS9-derived vector by *Pst*I/*Sma*I double digestion, gel-purified, and ligated into the corresponding *Pst*I/*Sma*I sites of all five constructs using T4 DNA ligase, generating the final versions of these vectors.

pYAB3:YAB3-SRDX，pTCP19:TCP19-SRDX and pTCP19:3HA-TCP19-SRDX constructs

To generate pYAB3:YAB3-SRDX, a 3,028-bp *YAB3* promoter fragment was amplified from Col-0 genomic DNA using primers incorporating *Pme*I and *PspX*I restriction sites. The purified PCR product was double-digested with *Pme*I and *PspX*I and ligated into the corresponding *Pme*I/*PspX*I-linearized pUBQ10:YAB3-SRDX backbone, thereby replacing the constitutive *UBQ10* promoter with the native *YAB3* promoter.

To generate pTCP19:TCP19-SRDX and pTCP19:3HA-TCP19-SRDX, two independent 4,015-bp *TCP19* promoter fragments were amplified from Col-0 genomic DNA, each with distinct terminal restriction sites. For pTCP19:TCP19-SRDX, the PCR product bearing *Sac*I and *PspX*I sites was double-digested with the corresponding enzymes and ligated into *Sac*I/*PspX*I-linearized pUB-DEST, generating the destination vector pTCP19-DEST, which was subsequently recombined with pDONR207-TCP19-SRDX via LR recombination (LR Clonase II, Invitrogen). For pTCP19:3HA-TCP19-SRDX, the PCR product bearing *Sma*I and *Nhe*I sites was digested with *Sma*I and *Nhe*I and ligated into *StuI*/*Spe*I-linearized pAtEXP7-3HA-DEST (laboratory stock) via compatible cohesive ends, yielding pTCP19-3HA-DEST, which was then recombined with pDONR207-TCP19-SRDX to produce the final construct.

pSPL8:SPL8-SRDX, pSPL8:YAB3-SRDX, and pSPL8:TCP19-SRDX constructs

To generate the *SPL8* promoter-driven dominant repression constructs pSPL8:SPL8-SRDX, pSPL8:YAB3-SRDX, and pSPL8:TCP19-SRDX, the corresponding *SPL8-SRDX*, *YAB3-SRDX*, and *TCP19-SRDX* fragments (synthesized by Sangon Biotech, Shanghai, China) were first recombined into the Gateway donor vector pDONR207 via BP reactions (BP Clonase II, Invitrogen), yielding the entry clones pDONR207-SPL8-SRDX, pDONR207-YAB3-SRDX, and pDONR207-TCP19-SRDX, respectively. Each verified entry clone was subsequently transferred into the laboratory-modified destination vector pAtSPL8(2,124-bp)-DEST via LR recombination (LR Clonase II, Invitrogen) to produce the final constructs.

pDRL1:SPL8-SRDX, pDRL1:YAB3-SRDX and pDRL1:TCP19-SRDX constructs

To generate pDRL1:YAB3-SRDX and pDRL1:TCP19-SRDX, a 2,004-bp *DRL1* promoter fragment bearing *Pme*I and *PspX*I sites was amplified from Col-0 genomic DNA. The purified fragment was double-digested with *Pme*I and *PspX*I and ligated into the corresponding sites of the previously generated pUBQ10:YAB3-SRDX and pUBQ10:TCP19-SRDX vectors,

respectively, thereby replacing the constitutive *UBQ10* promoter. To generate pDRL1:SPL8-SRDX, an independent 2,004-bp *DRL1* promoter fragment bearing *Sac*I and *Bgl*II sites was amplified and ligated into *Sac*I/*Bgl*II-digested pSPL8:SPL8-SRDX, replacing the *SPL8* promoter.

pREM22:YAB3-SRDX, pREM22:TCP19-SRDX, pA9:YAB3-SRDX and pA9:TCP19-SRDX constructs

To generate pREM22:YAB3-SRDX and pREM22:TCP19-SRDX, a 1,022-bp *REM22* promoter fragment with *Pme*I and *PspX*I sites was amplified from Col-0 genomic DNA, digested, and ligated into *Pme*I/*PspX*I-linearized pUBQ10:YAB3-SRDX and pUBQ10:TCP19-SRDX, replacing the *UBQ10* promoter. Similarly, a 1,461-bp *A9* promoter fragment was amplified with the same enzymes and ligated into the corresponding sites of the same linearized vectors to generate pA9:YAB3-SRDX and pA9:TCP19-SRDX.

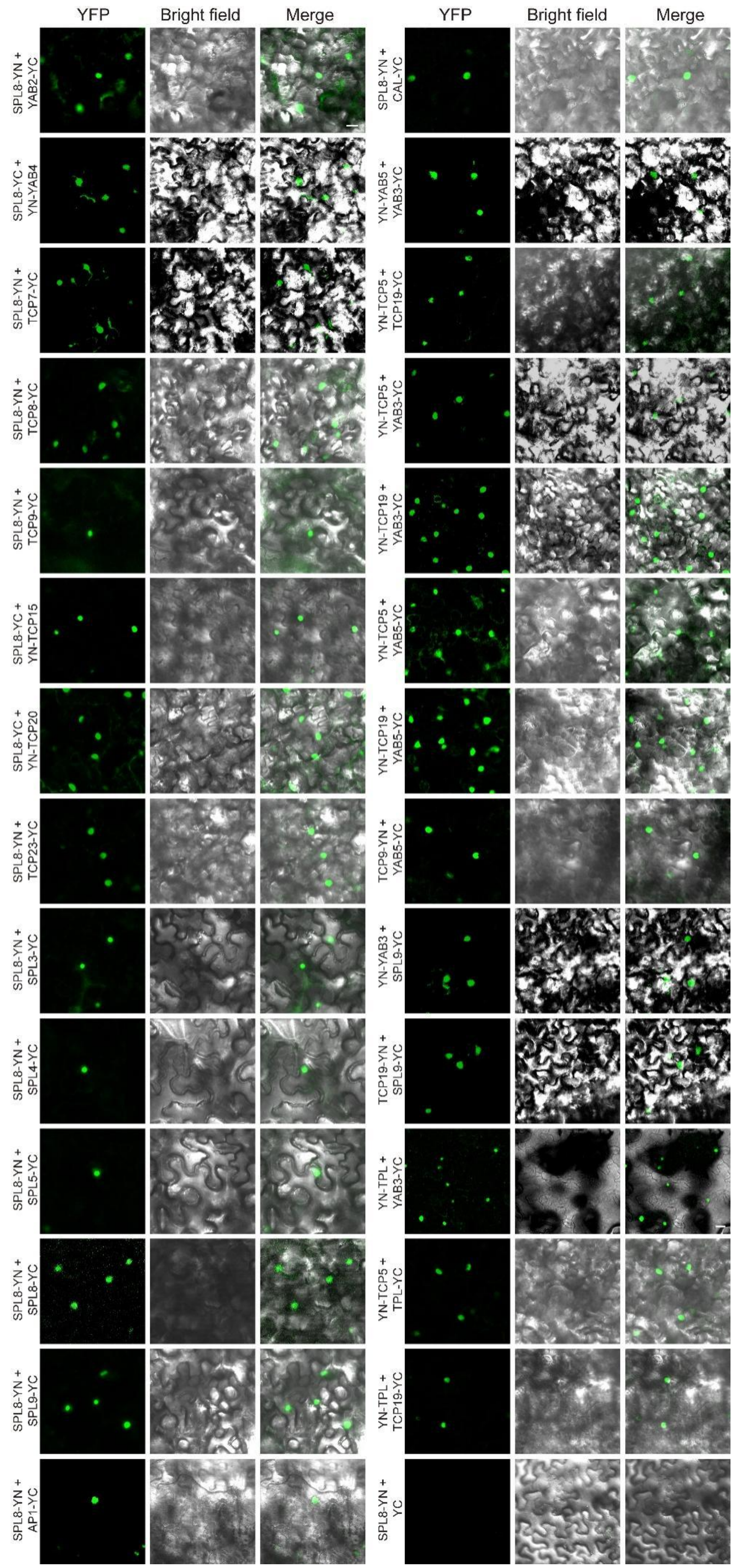
YFP
Bright field
Merge
YFP
Bright field
Merge
SPL8-YN + YAB2-YC
SPL8-YC + YN-YAB4
SPL8-YN + TCP7-YC
SPL8-YN + TCP8-YC
SPL8-YN + TCP9-YC
SPL8-YC + YN-TCP15
SPL8-YC + YN-TCP20
SPL8-YN + TCP23-YC
SPL8-YN + SPL3-YC
SPL8-YN + SPL4-YC
SPL8-YN + SPL5-YC
SPL8-YN + SPL8-YC
SPL8-YN + SPL9-YC
SPL8-YN + AP1-YC
SPL8-YN + CAL-YC
YN-YAB5 + YAB3-YC
YN-TCP5 + TCP19-YC
YN-TCP5 + YAB3-YC
YN-TCP19 + YAB3-YC
YN-TCP5 + YAB5-YC
YN-TCP19 + YAB5-YC
TCP9-YN + YAB5-YC
YN-YAB3 + SPL9-YC
TCP19-YN + SPL9-YC
YN-TPL + YAB3-YC
YN-TCP5 + TPL-YC
YN-TPL + TCP19-YC
SPL8-YN + YC

Figure S1. Protein–protein interactions validated via BiFC assay.

BiFC vectors were co-expressed in *Nicotiana benthamiana* leaves, and fluorescence signals were observed at 48 h post-infiltration. YFP fluorescence was detected for all tested protein pairs, indicating physical interactions. The combination of SPL8-YN and the empty YC vector served as the negative control. YN and YC represent the N- and C-terminal halves of YFP, respectively. Scale bars, 20 μm. Panel (YN-TPL+YAB3-YC) is presented at a different magnification, and its scale bar is indicated separately; the scale bar shown in panel (SPL8-YN+YAB2-YC) applies to all other panels.

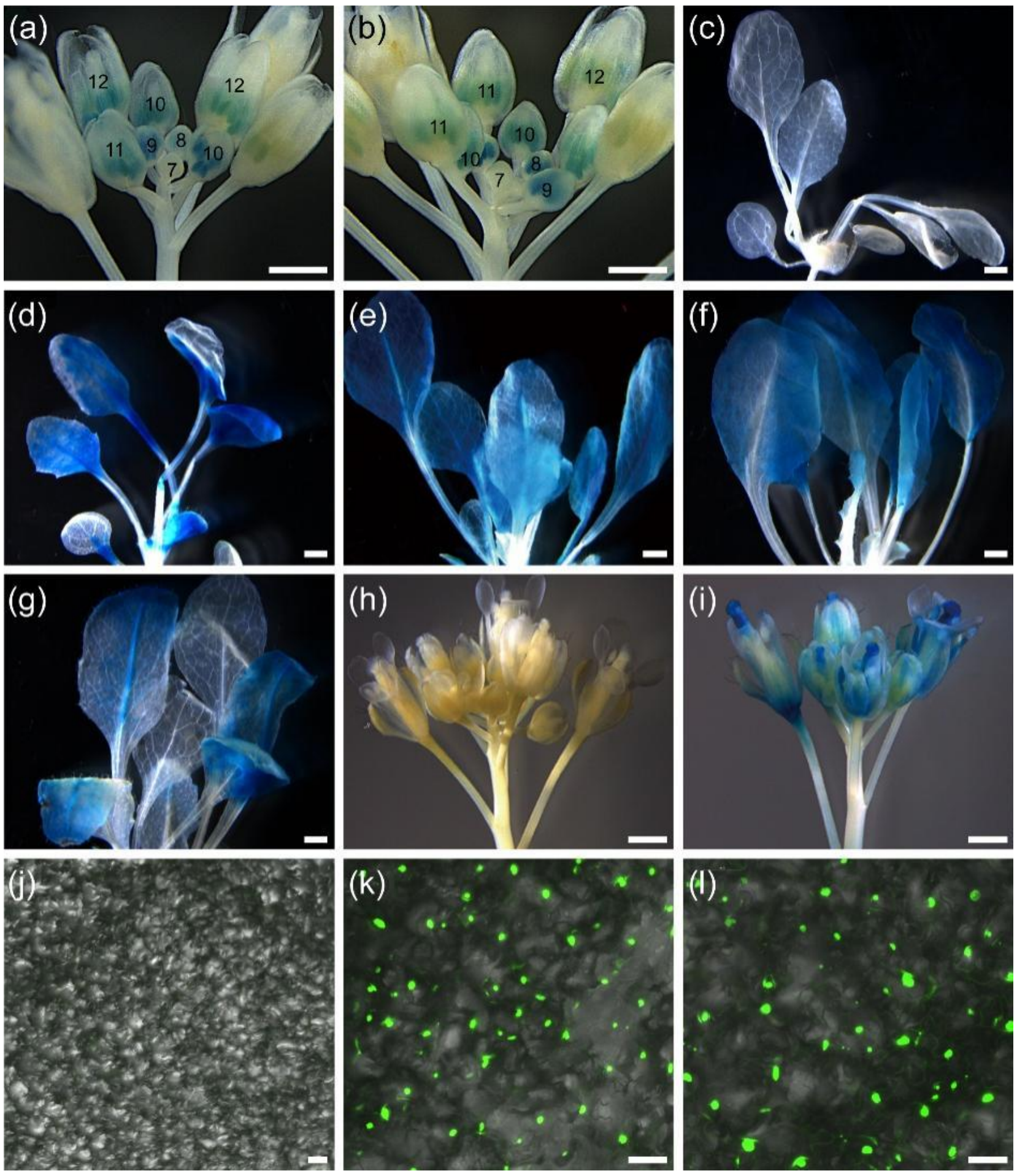

Figure S2. *DRL1*, *A9*, and heat-inducible *HSP18.2* promoter activities in GUS and GFP reporter lines

(a) GUS staining of *pDRL1:GUS* inflorescences.

(b) GUS staining of *pA9:GUS* inflorescences.

(c–i) GUS staining of *pHSP18.2:GUS* seedlings and inflorescences at various recovery time points after heat shock treatment, with non-heated controls.

(c–g) Seedlings: (c) Untreated control; (d–g) 2 h heat shock at 37 °C followed by recovery at 22–24 °C for 2, 4, 8, and 24 h, respectively.

(h,i) Inflorescences: (h) Untreated control; (i) 2 h heat shock at 37 °C followed by 6 h recovery at 22–24 °C.

(j–l) GFP signals in leaf epidermal cells of *pHSP18.2:SPL8-GFP* seedlings: (j) Untreated control; (k) 4 h recovery; (l) 10 h recovery at 22–24 °C after a 2 h heat shock at 37 °C.

Numbers in (a) and (b) indicate floral developmental stages. Scale bars: 1 mm for (a–i), 50 μm for (j–l).

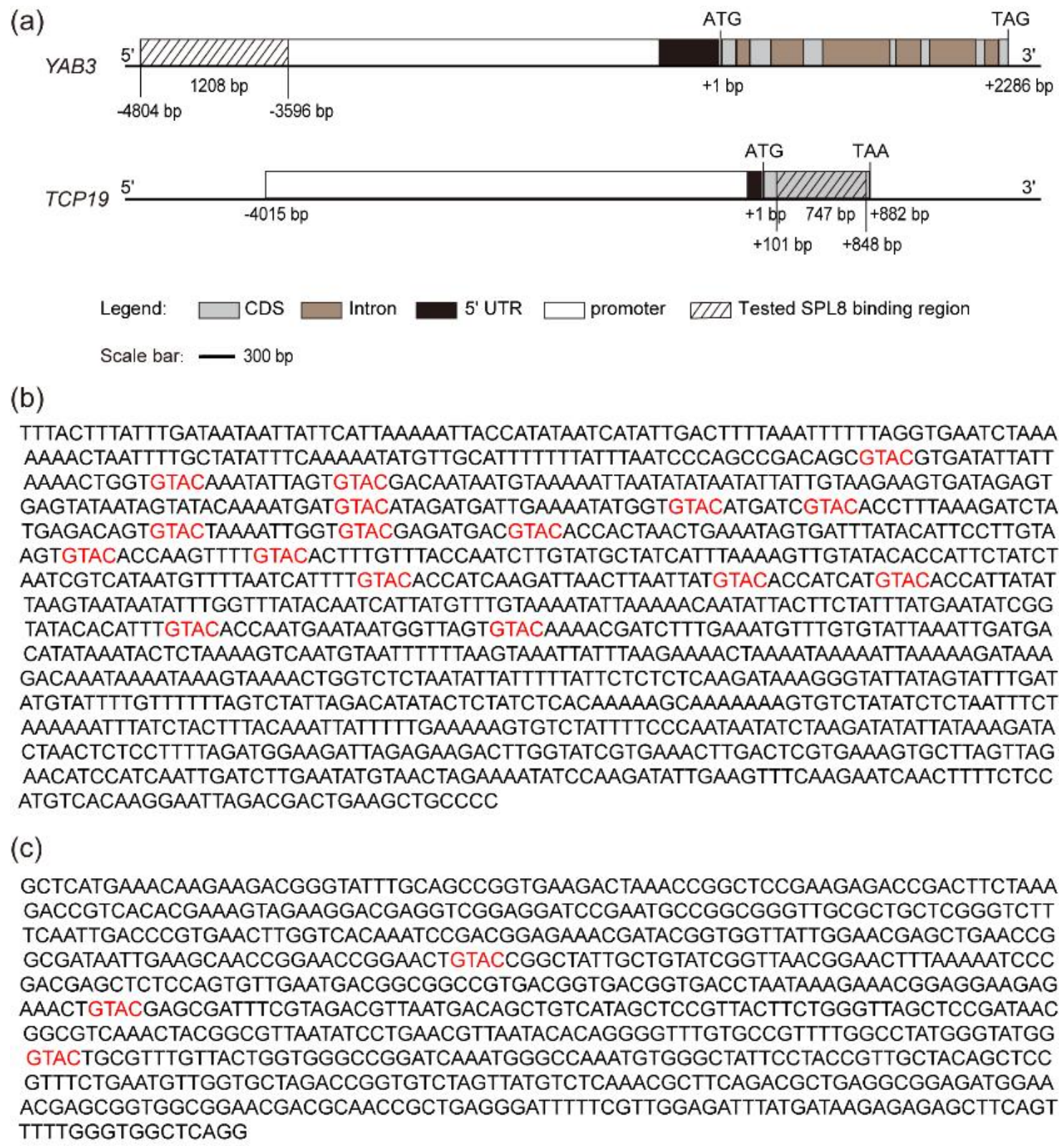

Figure S3. Gene structures and fragments used for yeast one-hybrid (Y1H) assays.

(a) Gene structures of *YAB3* and *TCP19*.

(b) 1,208-bp partial promoter fragment of *YAB3* for Y1H analysis.

(c) 747-bp partial coding sequence (CDS) fragment of *TCP19* for Y1H analysis.

SPL8 core-binding motifs (GTAC) are marked in red in (b) and (c).

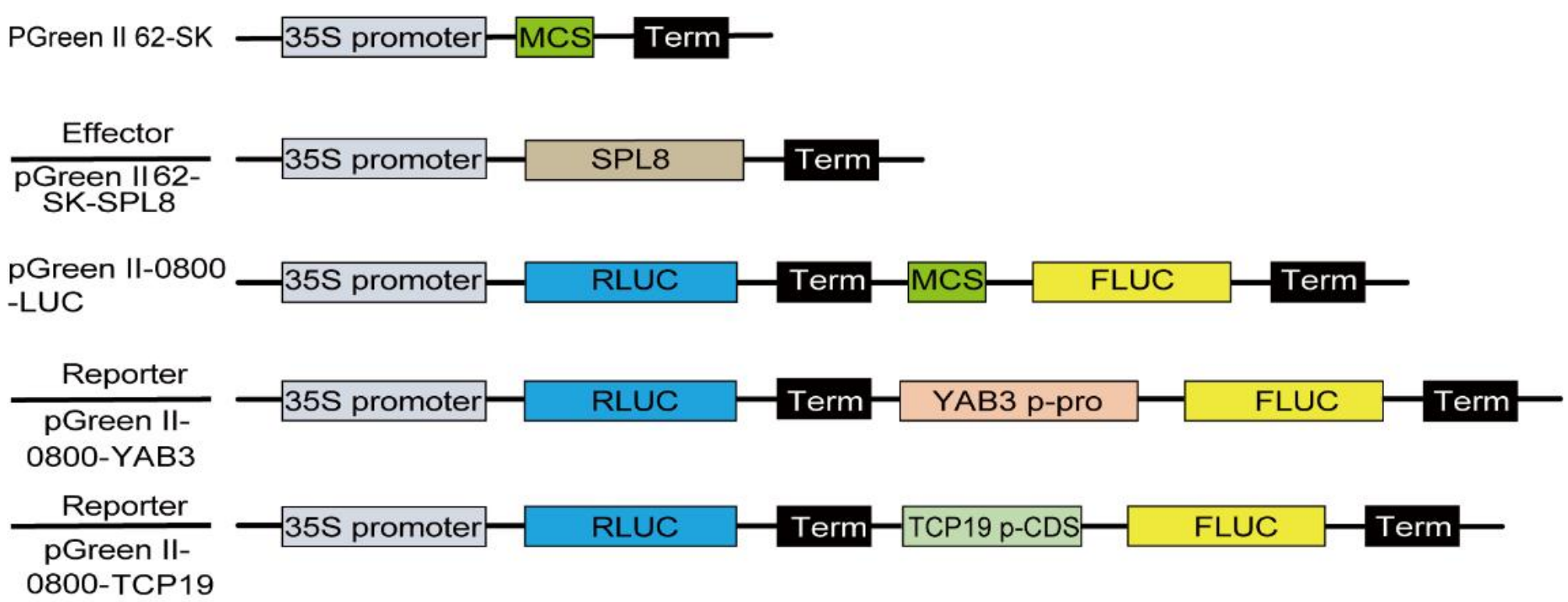

Figure S4. Schematic diagram of effector and reporter constructs for dual-luciferase assays.

The effector construct drives *SPL8* expression under the control of the CaMV 35S promoter. Reporter constructs contain the partial *YAB3* promoter (p-pro) or partial *TCP19* CDS (p-CDS) fused to *FLUC*, with *RLUC* serving as an internal reference control.

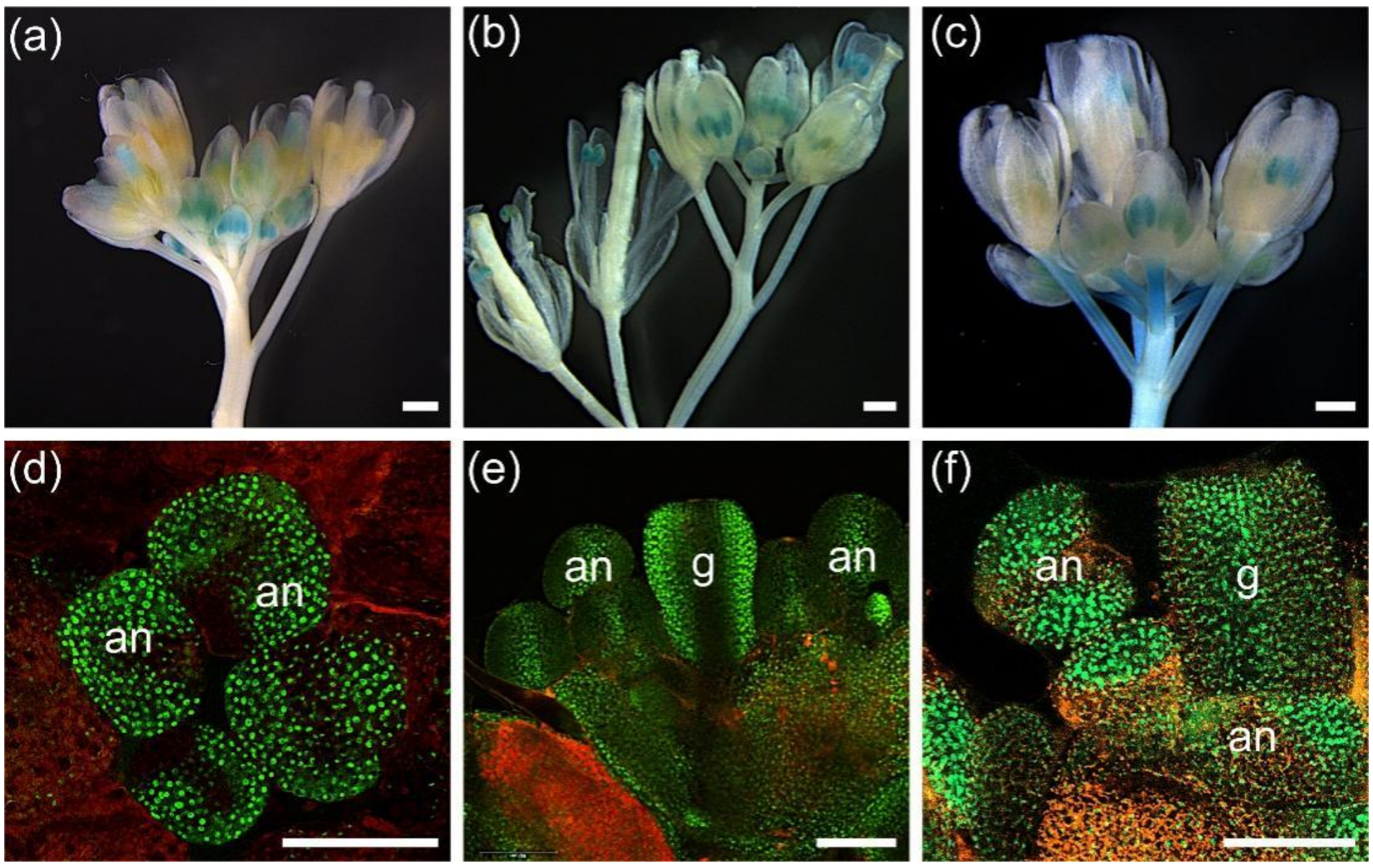

Figure S5. GUS reporter and GFP fusion analysis of *SPL8*, *YAB3,* and *TCP19* expression patterns in inflorescences.

GUS and GFP signals both indicate that *SPL8*, *YAB3*, and *TCP19* are expressed in anthers. (a–c) GUS staining in inflorescences of *pSPL8:GUS*, *pYAB3:GUS*, and *pTCP19:GUS* transgenic lines. (d–f) GFP fluorescence in *pSPL8:SPL8-GFP*, *pYAB3:YAB3-GFP*, and *pTCP19:TCP19-GFP* lines. an, anther; g, gynoecium. Scale bars, 0.5 mm for (a–c), 100 μm for (d–f).

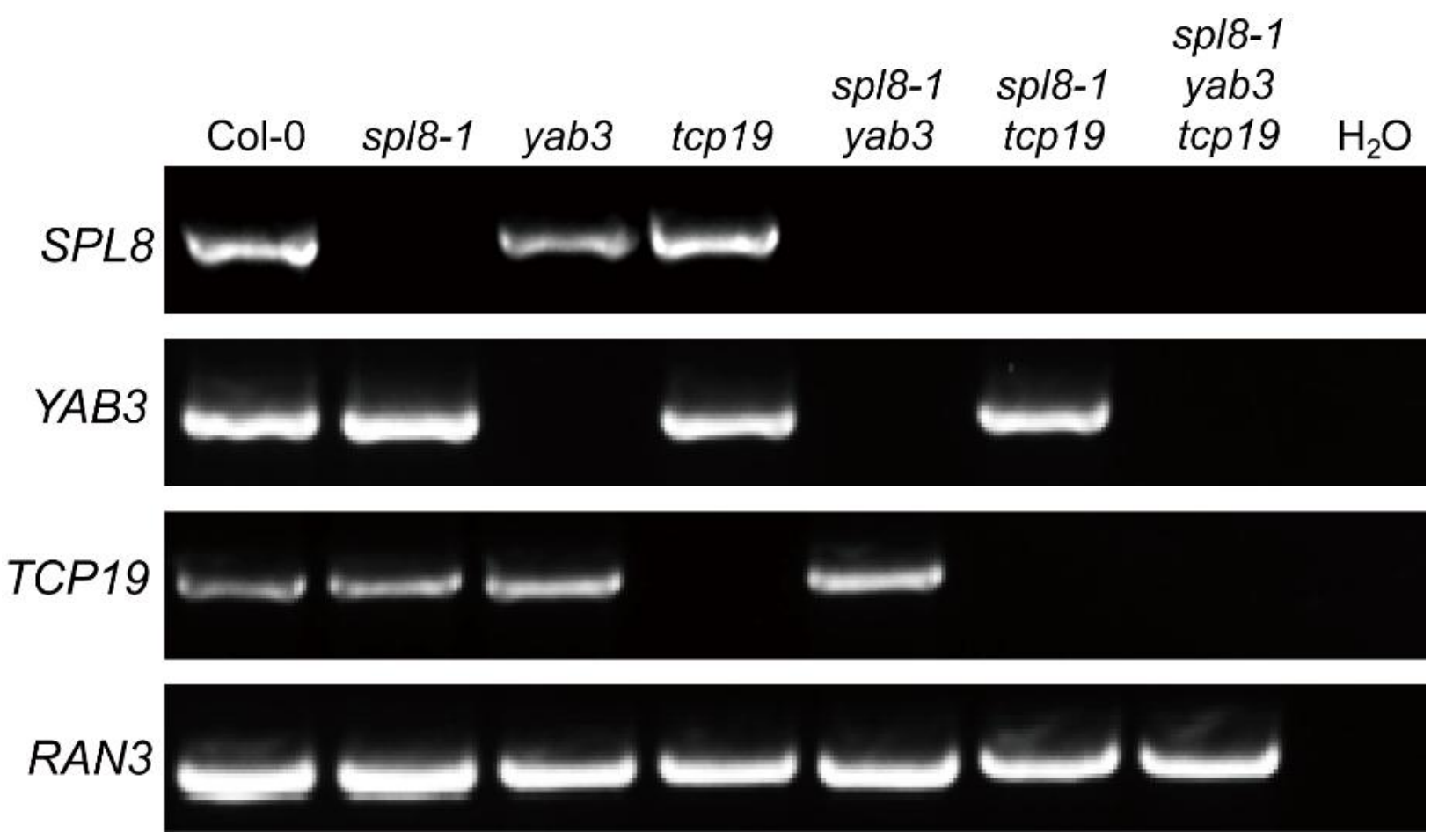

Figure S6. Expression analysis of *SPL8*, *YAB3*, and *TCP19* in different mutant backgrounds.

Total RNA was extracted from inflorescences of wild-type Col-0, single, double and triple mutants. RT-PCR analysis was performed to detect the transcript levels of *SPL8*, *YAB3*, and *TCP19* in the *spl8-1*, *yab3*, and *tcp19* single mutants, as well as their double and triple mutants. The results indicated that all mutant lines are null alleles. Water was used as a negative control.

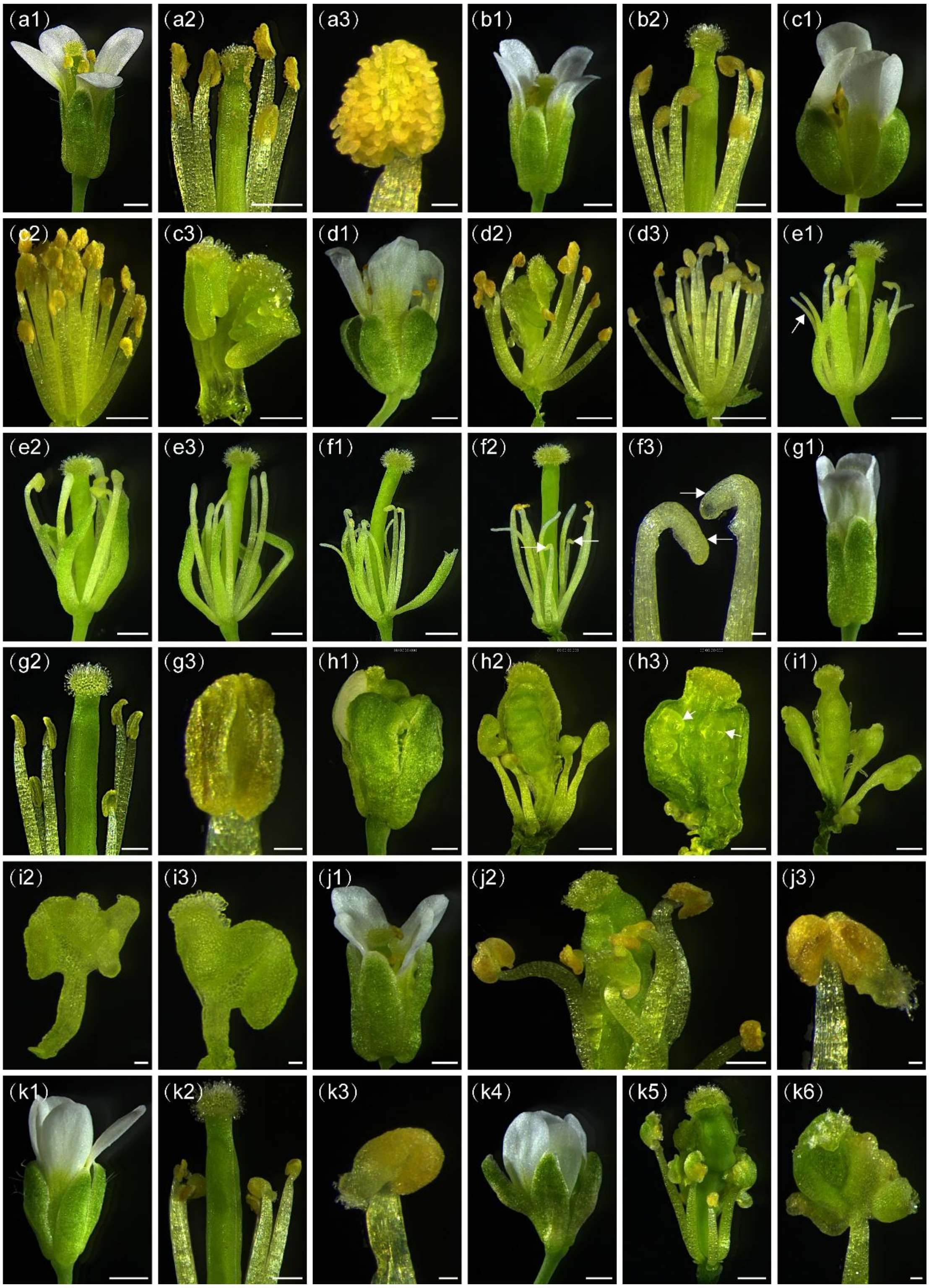

Figure S7. Floral phenotypes of various genotypes.

Flowers were dissected and observed after perianth removal.

(a1–a3) Wild-type Col-0. Abundant pollen grains are released from anthers and deposited on the stigma at anthesis (a2,a3).

(b1,b2) *spl8-1* mutant. No pollen grains accumulate on the stigma at anthesis (b2).
(c1–c3) *pUBQ10:YAB3-3HA* in the Col-0 background. Transgenic flowers exhibit slight swelling (c1), ectopic anther formation, and impaired gynoecium development (c2,c3).
(d1–d3) *pUBQ10:YAB3-3HA* in the *spl8-1* background. Flowers produce extra anthers with defective gynoecium development (d2,d3).
(e1–e3) *pYAB3:YAB3-SRDX* in Col-0. Transgenic plants display narrowed sepals, filamentous petals, reduced petal number, and clavate anthers (e1, arrow; e2,e3).
(f1–f3) *pYAB3:YAB3-SRDX* in *spl8-1*. Similar to the *pYAB3:YAB3-SRDX* in Col-0, flowers also produce filamentous petals and curved clavate anthers (arrows).
(g1–g3) *pA9:YAB3-SRDX* in Col-0. Anther pollen production is severely reduced, and almost no pollen grains are deposited on the stigma at anthesis (g2,g3).
(h1–h3) *pUBQ10:TCP19-SRDX* in *spl8-1*. Flowers fail to open at anthesis, with sepals enclosing most floral tissues (h1). Stamens are feminized, accompanied by enlarged and aberrant gynoecia (h2). Ovule structures are visible inside the ovary (h3, arrows).
(i1–i3) *pUBQ10:TCP19-SRDX* in Col-0. Feminized anthers are also frequently observed in transgenic flowers.
(j1–j3) *pTCP19:TCP19-SRDX* in Col-0. This genotype exhibits milder phenotypes than *pUBQ10:TCP19-SRDX* plants, yet abnormal feminized anthers are still detectable (j2,j3).
(k1–k6) *pREM22:TCP19-SRDX* in Col-0. Transgenic flowers show variable phenotypes, ranging from mildly feminized anthers (k2,k3) to severe defects with fully feminized anthers and aberrant gynoecium development (k5,k6).
Scale bars, 250 μm for (a2,b2,c3,h2,h3,i1,j2,k2,k5,g2), 50 μm for (a3,f3,g3,i2,i3,j3,k3,k6), and 500 μm for all other panels.

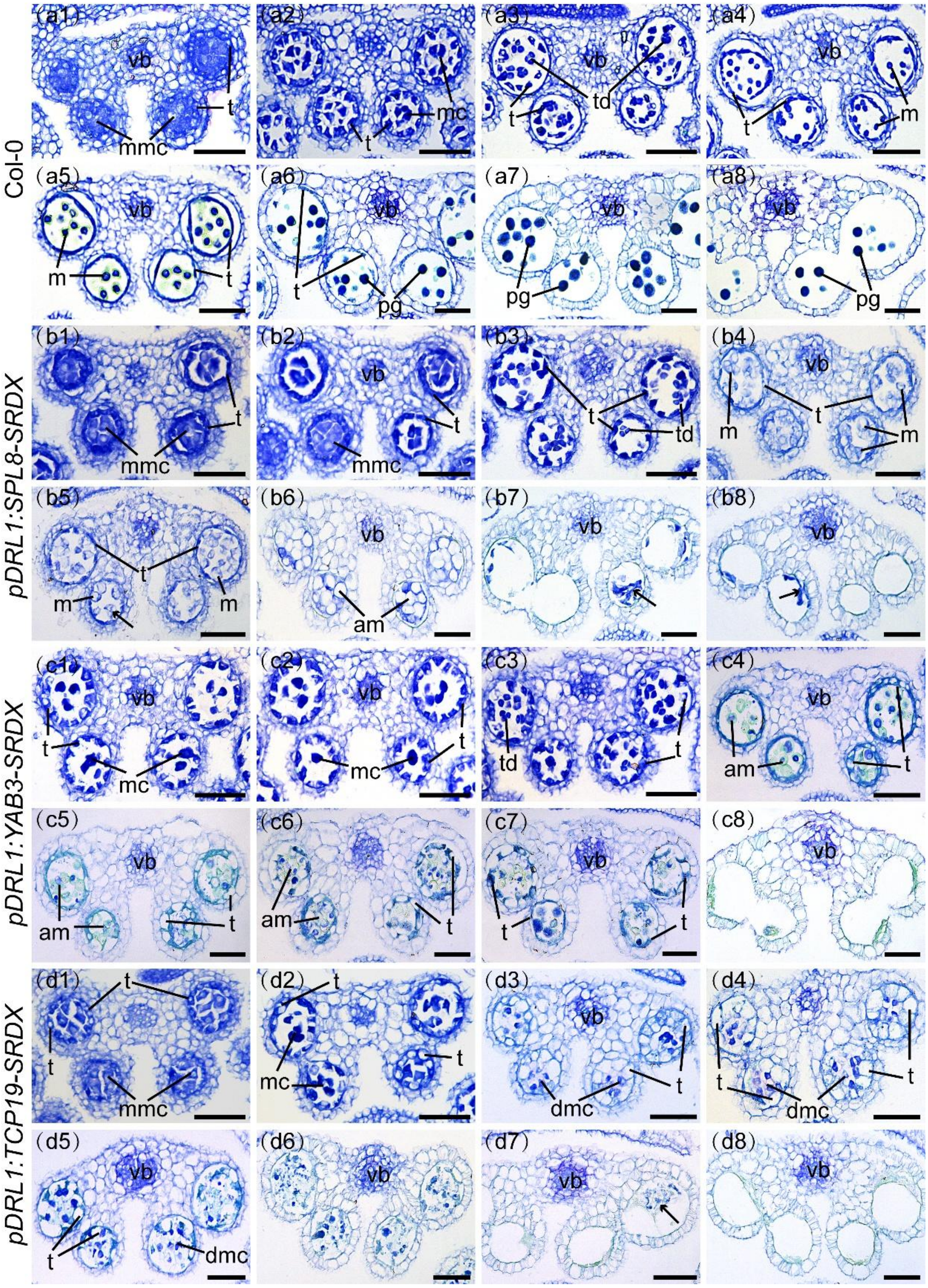

Figure S8. Anther histology of transgenic lines expressing SRDX fusions driven by the *DRL1* promoter.

(a1–a8) Wild-type Col-0.

(a1) Stage 5: anther containing microspore mother cells (MMCs).

(a2) Stage 6: differentiation of the tapetum layer surrounding meiotic cells (MCs).

(a3) Stage 7: formation of tetrads following meiosis.

(a4) Stage 8: release of microspores from tetrads.

(a5) Stage 9–10: normal microspore development with initial signs of tapetal degeneration.
(a6) Stage 11: formation of pollen grains, with persistent tapetal remnants still visible.
(a7) Stages 12: continued pollen maturation.
(a8) Stage 13: anther dehiscence and pollen release.
(b1–b8) *pDRL1:SPL8-SRDX* transgenic lines.
(b1,b2) Stage 5–6: the developing tapetum is clearly visible surrounding the MMCs and MCs.
(b3) Stage 7: tetrad formation proceeds normally.
(b4,b5) Stage 8–9: the majority of microspores fail to be released from the tetrads and remain aggregated as persistent tetrads.
(b6) Stage 10: microspores become highly vacuolated, filling the entire locules, while the tapetal layer completely disappears.
(b7,b8) Stage 12: the vacuolated microspores have almost entirely degenerated (arrowheads).
(c1–c8) *pDRL1:YAB3-SRDX* transgenic lines.
(c1,c2) Stage 6: some tapetal cells exhibit slight vacuolation, surrounding the centrally located MCs.
(c3) Stage 7: tetrad formation remains unaffected.
(c4) Stage 9: the tapetum displays pronounced vacuolation, concurrent with aberrant microspore development.
(c5) Stage 9–10: progressive vacuolation of tapetal cells is accompanied by the onset of microspore degeneration.
(c6,c7) Stage 10–11: degenerating microspores and tapetal remnants persist within the locules.
(c8) Stage 13: both microspores and the tapetum have completely degenerated, resulting in nearly empty locules.
(d1–d8) *pDRL1:TCP19-SRDX* transgenic lines.
(d1) Stage 5: differentiated tapetum surrounding MMCs, similar to the *pDRL1:YAB3-SRDX* transgenic lines.
(d2) Stage 6: tapetal cells exhibit slight vacuolation around MCs.
(d3) Stage 7–8: meiosis is completely arrested, with no tetrad formation; tapetal cells become enlarged and show pronounced vacuolation.
(d4) Stage 9: enlarged and highly vacuolated tapetum surrounds degenerating MMCs/MCs.
(d5,d6) Stage 10–11: the locules are filled with degenerating tapetal and MMC/MC remnants.
(d7,d8) Stage 12: residual degradation products persist within the locules (arrowhead), with complete degeneration of all cellular contents also observed, resulting in empty locules.
Abbreviations: am, abnormal microspore; dm, degenerated microspore; dmc, degenerating meiotic cell; m, microspore; mc, meiotic cell; mmc, microspore mother cell; pg, pollen grain; t, tapetum; td, tetrad; vb, vascular bundle. Scale bars, 50 μm.

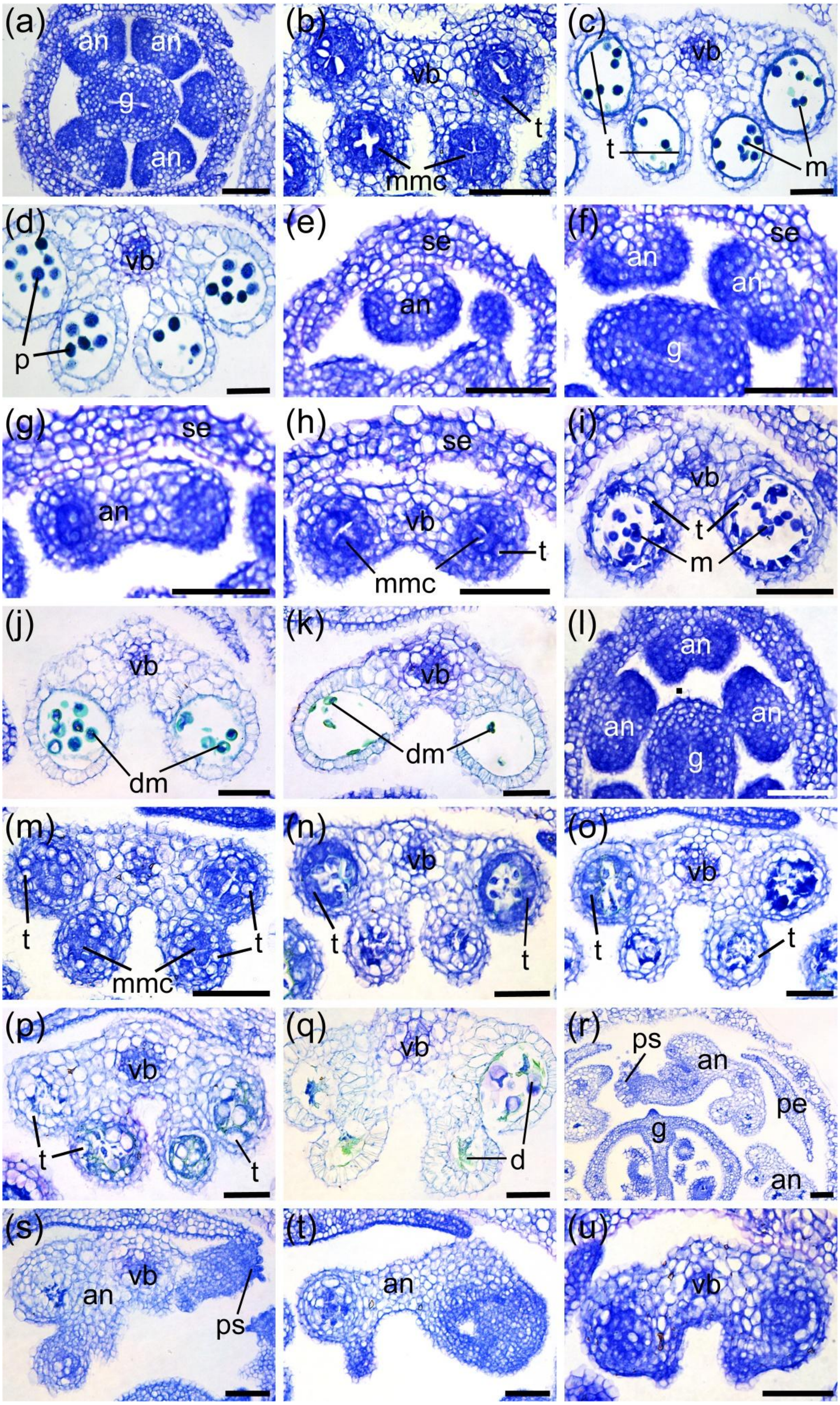

(a)
an
an
g
an
(b)
vb
t
mmc
(c)
vb
t
t
m
(d)
vb
p
(e)
se
an
(f)
an
se
an
g
(g)
se
an
(h)
se
vb
mmc
t
(i)
vb
t
m
(j)
vb
dm
(k)
vb
dm
(l)
an
an
an
g
(m)
t
t
mmc
t
(n)
vb
t
t
(o)
vb
t
t
(p)
vb
t
t
t
(q)
vb
d
(r)
ps
an
pe
g
an
(s)
an
vb
ps
(t)
an
(u)
vb

Figure S9. Histological analysis of anther cross-sections of transgenic lines expressing *YAB3-SRDX* or *TCP19-SRDX* under the *REM22* promoter.

(a–d) Wild-type Col-0. Anthers show normal development at stage 3 (a). At stage 5 (b), microspore mother cells are enclosed by the tapetum and surrounding anther wall layers. After meiosis, microspores develop normally, giving rise to pollen grains by stage 11, with tapetal remnants still present (c), and pollen grains continue to mature through stage 12 (d).

(e–k) *pREM22:YAB3-SRDX* in Col-0. The most prominent defect is the formation of only two pollen sacs per anther, a phenotype that originates at early developmental stages, as seen at stages 3–5 (e–g). Meiosis proceeds normally through stage 6 (h) to form tetrads at stage 7 (i), although obvious tapetal vacuolation is already evident. Thereafter, from stage 10 through stage 13, microspores undergo progressively degeneration, resulting in nearly empty locules at anthesis.

(l–u) *pREM22:TCP19-SRDX* in Col-0. Early anthers show normal morphology at stage 3 (l). At stage 5 (m), the tapetum surrounding the microspore mother cells becomes vacuolated. During meiotic stages (stages 6–7), tapetal cells enlarge; some remain densely cytoplasmic without evident vacuolation, while others become highly vacuolated (n,o). Concurrently, microspore mother cells and meiotic cells fail to complete meiosis and degenerate. By stage 10–11 (p), the tapetum exhibits severe vacuolation and eventually degenerates by stage 13 (q). Additionally, some anthers develop feminized structures (r,s) and reduced pollen sacs (t,u).

Abbreviations: an, anther; dm, degenerated microspore; dmc, degenerated meiotic cell; g, gynoecium; mc, meiotic cell; mmc, microspore mother cell; pg, pollen grain; ps, pistil-like structure; se, sepal; t, tapetum; vb, vascular bundle.

Scale bars, 50 μm.
